# Discharge Competence and Pattern Formation in Peatlands: A Meta-Ecosystem Model of the Everglades Ridge-Slough Landscape

**DOI:** 10.1371/journal.pone.0064174

**Published:** 2013-05-09

**Authors:** James B. Heffernan, Danielle L. Watts, Matthew J. Cohen

**Affiliations:** 1 Nicholas School of the Environment, Duke University, Durham, North Carolina, United States of America; 2 School of Natural Resources and Environment, University of Florida, Gainesville, Florida, United States of America; 3 School of Forest Resources and Conservation, University of Florida, Gainesville, Florida, United States of America; DOE Pacific Northwest National Laboratory, United States of America

## Abstract

Regular landscape patterning arises from spatially-dependent feedbacks, and can undergo catastrophic loss in response to changing landscape drivers. The central Everglades (Florida, USA) historically exhibited regular, linear, flow-parallel orientation of high-elevation sawgrass ridges and low-elevation sloughs that has degraded due to hydrologic modification. In this study, we use a meta-ecosystem approach to model a mechanism for the establishment, persistence, and loss of this landscape. The discharge competence (or self-organizing canal) hypothesis assumes non-linear relationships between peat accretion and water depth, and describes flow-dependent feedbacks of microtopography on water depth. Closed-form model solutions demonstrate that 1) this mechanism can produce spontaneous divergence of local elevation; 2) divergent and homogenous states can exhibit global bi-stability; and 3) feedbacks that produce divergence act anisotropically. Thus, discharge competence and non-linear peat accretion dynamics may explain the establishment, persistence, and loss of landscape pattern, even in the absence of other spatial feedbacks. Our model provides specific, testable predictions that may allow discrimination between the self-organizing canal hypotheses and competing explanations. The potential for global bi-stability suggested by our model suggests that hydrologic restoration may not re-initiate spontaneous pattern establishment, particularly where distinct soil elevation modes have been lost. As a result, we recommend that management efforts should prioritize maintenance of historic hydroperiods in areas of conserved pattern over restoration of hydrologic regimes in degraded regions. This study illustrates the value of simple meta-ecosystem models for investigation of spatial processes.

## Introduction

Regular self-organized spatial patterning of ecological systems results from spatially-dependent feedbacks whose strength and sign vary with distance [1]. While strong local feedbacks can generate discrete patches on the landscape [2], [3], regular patterning of such mosaics is generated by distal negative feedbacks, by which the presence of organisms at one location inhibits their establishment or persistence at some distance [4]. In some cases, organisms concentrate a limiting resource such as water or soil nutrients [5]–[8], facilitating local expansion while limiting suitability of locations outside of occupied patches. In others, plants or animals may locally mitigate a stressor such as temperature, salinity, or shear stress but exacerbate the stressor outside the area of biotic influence [9], [10]. The diversity of potential distal feedbacks in any given ecosystem requires the development of distinctive predictions that discriminate among plausible mechanisms [11], [12].

When distal negative feedbacks are coupled to strong local positive feedbacks, spatial patterning can exhibit global bi-stability, meaning that alternative equilibria may exist at the scale of entire landscapes [13]. In such cases, either regular patterning or unstructured, homogenous states may exist under the same set of environmental conditions. Transitions between patterned and homogenous states may be sudden, and trajectories of recovery may exhibit hysteresis (meaning that transitions in one direction occur at a different threshold in conditions than transitions in the other direction [11], [13]). While some researchers have argued that regular spatial patterning always exhibits global bi-stability across some range of conditions [13], others have illustrated that regular patterning can arise via stochastic processes that do not produce such landscape-scale transitions [14], [15]. Nonetheless, the potential for hysteretic responses of patterning to environmental drivers presents a challenge to their conservation and restoration [16]–[18], particularly since the loss of patterning can have important implications for ecosystem function [13], [19] and habitat value [20].

Patterned landscapes are most common in wetlands and drylands, where the presence of water exerts strong control over vegetation, but where biotic feedbacks on water storage and movement are also strong [11], [21], [22]. These systems exhibit a wide range of geometries, including isolated patches, strands, and rings, that arise from the specific characteristics of biotic and abiotic feedbacks, including their strength and spatial dependency [11], [23]–[25]. While many patterned landscapes are isotropic, others exhibit characteristic directionality or orientation of pattern features [26]. For example, in string fens, flow-perpendicular orientation of woody vegetation is thought to reflect anoxic stress caused by impoundment behind raised soil strands [6], [27]. In arid lands, banded vegetation such as the Tiger Bush landscape in the Sahel is thought to arise from trapping of runoff and associated nutrients [24], [28]–[30]. The spatial orientation of patches in such landscapes reflects differential operation of spatially-dependent feedbacks with direction; in other words, local positive or distal negative feedbacks differ in their strength depending on direction, with stronger positive or weaker negative feedbacks operating in the direction of patch elongation [11], [22], [25].

Because the spatial interactions that generate and maintain patterned landscapes are complex, dependence of pattern formation on conditions or parameters is frequently assessed using simulation models. The benefit of this approach is that the spatially explicit outcomes can be compared to observed pattern, potentially using a variety of metrics. However, because complex models cannot be formally solved, the bounds on model behavior (e.g., parameter combinations that support emergence of appropriate pattern, occurrence of global bi-stability) must be assessed via statistical analysis of model outputs. While simple spatial models lack the ability to reproduce spatial patterns explicitly, they can provide a broader understanding of feedback behavior by allowing global, closed-form solutions for equilibria [31], . Such solutions can identify novel relationships and dynamics that broaden the suite of testable predictions available for evaluating proposed mechanisms.

Relatively simple analytical models have been recently used to assess the dynamics of resource exchange, productivity, and food web structure in spatially-coupled (or meta-) ecosystems [32]–[34], but this approach has not been applied to the physical exchanges of water to understand biogeophysical feedbacks in wetlands [34]. Current models of wetland pattern formation incorporate the presence of surface water, but rarely address surface flow explicitly (e.g., [6], [11]; but see [25], [XPATH ERROR: unknown variable "next".]). In this study, we use a simple meta-ecosystem model [35], [36] to evaluate ecohydrologic feedbacks between wetland microtopography, soil accretion, and water flow in peatlands, with an emphasis on the elongated patterning in the ridge-slough mosaic of the Everglades.

### Florida Everglades Ridge-Slough Mosaic

**Figure 1:**
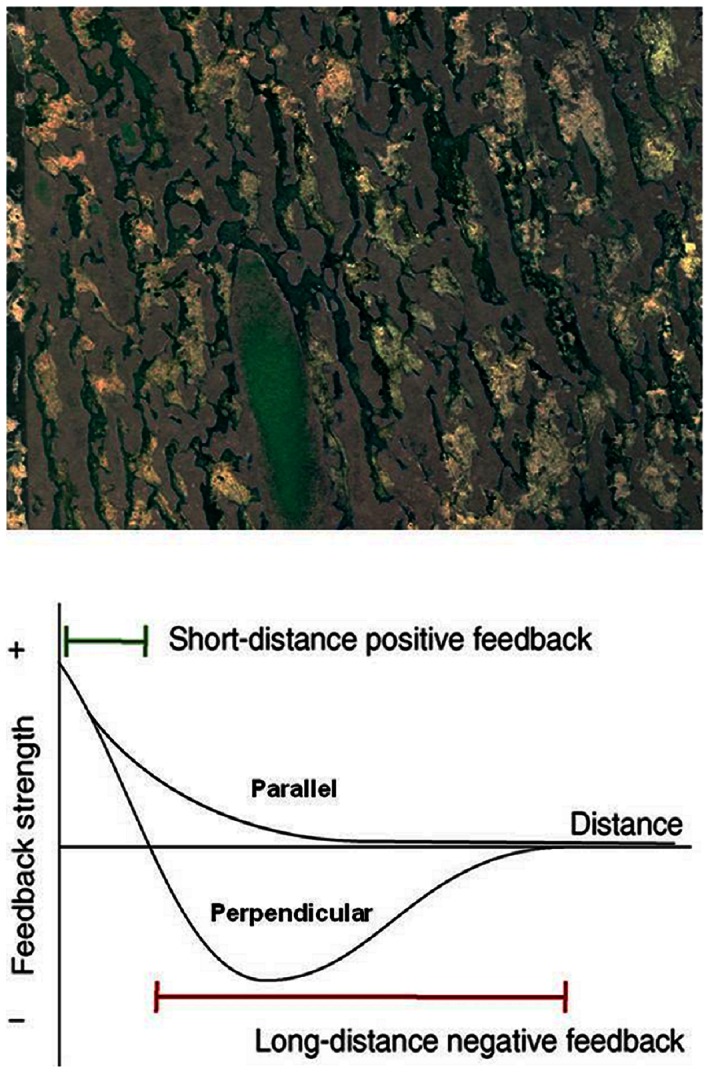
**Flow-oriented patterning in the Ridge and Slough landscape of the Florida Everglades (top) is hypothesized to arise from coupled spatial feedbacks that operate anisotropically (i.e.differently depending on direction; bottom panel).** Higher elevation ridges support dense stands of sawgrass (*C. jamaicense;* green patches in A,B; grey patches in C), and are interspersed in a matrix of sloughs that support floating and submerged vegetation (dark green in C) and floating periphyton mats (light patches in C). The differentiation of these patches is hypothesized to reflect local positive feedbacks among vegetation, peat production, soil elevation, and hydroperiod (bottom panel). The pronounced linearity of these features suggests that the strength of distal negative feedbacks is stronger in the flow-parallel than in the flow-perpendicular direction. This study presents a model of such feedbacks that arise from the differential capacity of patches to convey water, and the consequences of that variation in discharge competence for hydrologic regimes across the landscape.

The ridge-slough landscape of the central Everglades is a lotic peatland in which distinct vegetation assemblages are organized by microtopographic variation and associated differences in hydroperiod [37], [38]. Higher elevation ridges experience annual hydroperiods of 300–340 days [39]–[41], and are occupied by largely monotypic stands of sawgrass (*Cladium jamaicense*); sloughs contain a mixture of floating and submerged plants and calcareous periphyton mats that thrive under continuous inundation. Both patch types have characteristic widths of 50–150 m; sloughs are generally, but not universally, continuous (i.e., longitudinally connected in the direction of flow), while ridges vary considerably in length [40], [42], [43], creating a landscape pattern elongated in the direction of historical flow (Fig 1). Prior to modern hydrologic changes, elevation differences between ridges and sloughs are estimated to have ranged from 30 to 60 cm [41], [44]. Intermediate depths between ridges and sloughs were relatively rare historically [41] and in conserved landscapes [40], but support a more diverse assemblage of emergent species, in a community commonly referred to as wet prairie [37]–[39]. The abundance of the wet prairie community varies considerably across the landscape [39], [40] and increases during dry periods when slough hydroperiods and inundation depths decline [38], [45]. Tree islands occupy a smaller portion (2–3%) of this landscape [3], [41].

Historically, the ridge-slough mosaic accounted for more than two thirds of the Everglades landscape [41], but the present distribution has been substantially reduced by urban development to the east, and agriculture to the north [46]. Within the current landscape, hydrologic modification associated with historic drainage and impoundment by canals has led to degradation of historic landscape pattern, in terms of both the horizontal structure of vegetation [42], [43] and microtopographic relief [40]. Other changes include phosphorus-driven eutrophication and cattail encroachment downstream of canals [47], [48], and alterations of fire frequency and intensity associated with landscape drainage [49], [50].

Preserving and re-establishing historic landscape structure is a central goal of Everglades restoration [51]–[53]. The existence and structure of the ridge-slough mosaic is itself viewed as a fundamental characteristic of this landscape, but the spatial patterning of soil elevation (and thus water depth) and vegetation also has important implications for charismatic fauna such as wading birds and their prey [20]. During dry periods, deeper portions of the landscape provide refugia for fish and other obligate aquatic organisms; dry-season concentrations of prey in sloughs also provides abundant food and easy foraging for wading birds and other predators. Understanding the mechanisms by which this landscape established and persists is thus crucial if a wide range of restoration goals are to be achieved.

### Mechanisms of ridge-slough pattern formation

Given the orientation of ridges and sloughs, it is widely agreed that flow plays an important role in producing their spatial pattern. However, the mechanism by which flow induces patch linearity remains unresolved. The hydraulic focusing hypothesis proposes that differential evapotranspiration between ridges and sloughs drives accumulation of phosphorus and thus greater productivity of ridges [54], [55]. Phosphorus may accumulate in tree islands by this mechanism [56]–[59], but evidence for such hydraulic gradients between ridges and sloughs is limited.

Differential sediment deposition was suggested initially as a plausible and even likely explanation for both ridge-slough elevation differences and landscape pattern [51], [60]. More recently, Larsen and Harvey [25], [26] presented a simulation model (RASCAL) that generates ridge-slough-like patterns via differential sediment transport, based on differences in velocity in ridges and sloughs under high water slope conditions. While this remains a plausible explanation, several assumptions of this hypothesis, including the suitability of flocculent organic matter to produce peat and the historic frequency of high flow velocities, remain unverified [22].

The discharge competence (or self-organized canal) hypothesis proposes that feedbacks among local peat accretion, microtopography, and hydroperiod favor development of connected sloughs that act essentially as canals, whose greater capacity to convey water (i.e. discharge competence) more efficiently drains water from the landscape and thus controls hydroperiod [22], [40], [61]. At the local scale, ridges and sloughs are hypothesized to be alternative ecosystem configurations with the same (slightly positive) local net carbon balance: higher elevation ridges support greater productivity, but also greater soil respiration, while sloughs support less productive vegetation communities but smaller gross C losses to oxidation. These local feedbacks are linked to spatial pattern by flow that arrives at the upstream landscape boundary, and must be conveyed over the peat surface [39]. Ridges and sloughs differ substantially in depth [40], [41] and somewhat in vegetation friction (i.e., flow resistance) [62]–[64], creating marked differences in their competence to route water. Given finite upstream flows, increased abundance of sloughs will, according to this hypothesis, lead to lowered water levels, and thus to conditions favoring ridge expansion; conversely, ridge expansion will tend to increase water levels particularly when flow connectivity is impeded, creating hydroperiod conditions more favorable to slough expansion [22]. Importantly, in order for this distal negative feedback to generate linear, flow-parallel features, it must act anisotropically (i.e. differently depending on direction). Ridge expansion parallel to flow would thus have only a weak influence on water levels, whereas ridge expansion perpendicular to flow would more severely reduce the cross-sectional area and, ultimately, connectivity of sloughs available to convey water [22]. Some models suggest more generally that landscape pattern arises to optimize some particular ecological function [65]. Given the poor capacity of the low-relief Everglades landscape to shed water, we argue that the organization of the ridge and slough landscape around discharge capacity represents a plausible primary driver of landscape pattern.

Our meta-ecosystem model [35] describes interactions among local C balance, elevation, and discharge competence (i.e., the capacity of a flow-perpendicular cross-section to convey water). Our objectives in developing this formal model of the self-organizing canal hypothesis are threefold. First, we sought to determine whether feedbacks between C balance and water depth are sufficient to produce local attractors in absolute and relative depth (and thus vertical divergence of ridges and sloughs). Second, we sought to assess the influence of water displacement and flow (or their absence) on these feedbacks, with the end goal of assessing their ability to produce the anisotropic (directional) distal feedbacks necessary to produce the elongation characteristic of the ridge-slough mosaic. In other words, this model is intended to assess the sufficiency of the discharge competence (or self-organizing canal) hypothesis to produce the observed geometry of the ridge slough landscape in the absence of sediment or nutrient redistribution. Third, we sought to determine the potential for global bi-stability in the ridge-slough landscape (i.e. where the ridge-slough mosaic and a topographic homogeneity represent alternative states at the landscape scale). This last objective has important implications for the conservation and restoration of the ridge-slough landscape. In contrast with other models of the ridge-slough landscape that rely on numerical simulations, the relatively simple analytical model presented here can be solved, allowing for more complete assessment of solutions and consideration of alternative relationships.

## Model Structure and Analysis

### Local C balance

Our model begins with the mass balance of peat accumulation and resulting changes in soil elevation (z), which is based on the difference between organic matter production by vegetation (P) and decomposition of organic matter (R):
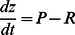
(1)Eq. 1 implicitly defines productivity and respiration in dimensions of length (i.e. as vertical soil accretion rather than areal C stock per se), and represents the long-term accumulation and loss of organic matter over a homogenous area of undefined extent. Our goal is to assess the sufficiency of the discharge competence mechanism to produce local positive and distal negative feedbacks, rather than to model Everglades C budgets per se. Thus we ignore other mechanisms of OM redistribution such as dissolved OM export, which is a small fraction of net C budgets [66], and particulate C exchange, which has already been shown in principle to be able to create such feedbacks [26]; we also do not explicitly incorporate soil loss due to fire.

Feedbacks between soil elevation and C balance (and thus soil accumulation) emerge because both respiration and production depend on mean water depth. Initially, we define water depth (D) as the difference between soil elevation (z) and the long term mean water level (h).
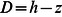
(2)so that increases in elevation leads to decreases in depth.

Net primary productivity (NPP) of ridges is generally higher than that of submerged and floating-leaved macrophytes in sloughs [47], [67]–[69]. Moreover, while sawgrass production decreases with greater water depth, it also declines where water depths are extremely shallow (D.L. Childers, unpublished data). Estimates of calcareous periphyton production are extremely variable, but generally below that of sawgrass [70], and the highly organic character of soil in the ridge-slough mosaic [71] suggests that mineral calcium carbonate accumulation contributes negligibly to soil accretion. The lability of organic matter produced by periphyton communities in general suggests that the organic contribution of these autotrophs to soil development is likely smaller than that of more recalcitrant sawgrass, although biomarker studies indicate that some algal organic matter is incorporated into Everglades peat [63], [72]. Together, these patterns contribute to the hypothesized depth-dependence of primary production and potential peat accumulation that underlie several models of ridge-slough pattern formation [22], [39], [60].

**Figure 2:**
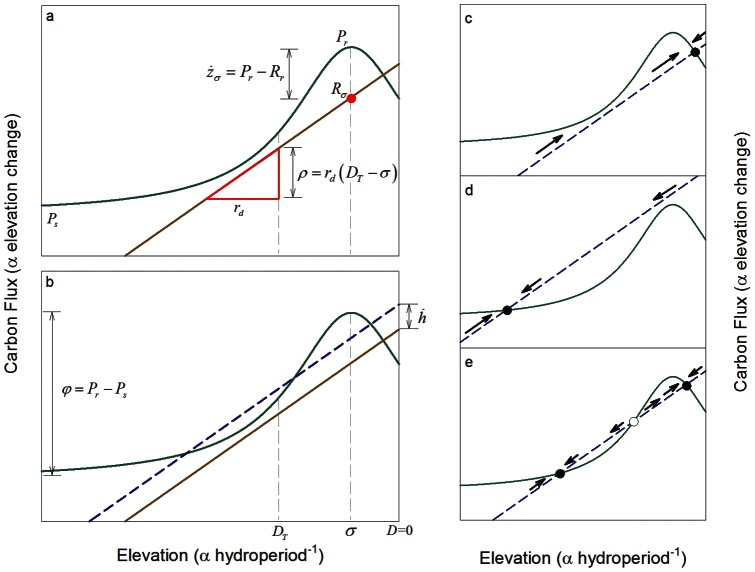
**Relationships between water depth and carbon balance (and thus soil accretion) in the model peatland patches.** (a) Soil accretion is driven by the balance of organic matter production (P; green line) and decomposition (R; brown line). Primary production has a maximum value of P_σ_ at depth σ; net soil accretion at that optimum (z_σ_) is the difference between P_r_ and respiration rates at the depth of maximum production (R_σ_). Primary production declines to the mean of ridge (P_r_) and slough (P_s_) production at D_T._ Respiration declines with depth with a slope of r_d_. The derived parameter ρ is the change in respiration over the vertical distance (D_T_ – σ). (b) The derived parameter φ is the difference between ridge (P_r_) and slough (P_s_) productivity. Changes in water level 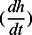 modify the depths at which soil accretion maintains stable depths. Depth equilibria (c–e) occur at water depths where changes in elevation of soil and water surface are equal. Depending on the values of parameters, depth equilibria (solid symbols) may occur at relatively (c) shallow or (d) deep water levels, or (e) both, in which case the intermediate equilibrium (open symbol) is unstable (i.e., a threshold).

We model primary production as a function of water depth. In this model, gross primary production (P) has a maxima at the long-term mean water depth that is optimum for sawgrass growth (σ), and declines with increasing and decreasing depth (Fig 2A):
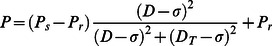
(3)where *P*_s_ is the gross peat production in sloughs, *P*_r_ is gross peat production at the optimal depth for sawgrass growth, and *D_T_* is the depth at which peat accretion is the average of these two end-members (analogous to a half-saturation constant in monod kinetics). It is important to note that the equilibrium analysis developed from this model rests on the assumption that the response variable (in this case, peat accretion) changes much more rapidly than the driver variable (in this case, long-term mean water depth). Thus, Eq. 3 should be interpreted as describing a relationship between the long-term mean (i.e., over timescales of decades to centuries) of water depth and of gross peat production, rather than the instantaneous level and rate, respectively. Of course, primary production and water depth vary considerably over seasonal and inter-annual time scales, and short-term relationships between water depth and productivity may depend on plant community composition; Eq. 3 integrates that variation over timescales of soil accumulation and loss, over which the relationship between peat production and mean water depth is presumed to be relatively smooth and continuous. Our model does not explicitly address the role of woody vegetation establishment in relationships between water depth and productivity, but similar threshold changes in productivity are likely under drier conditions that allow for tree recruitment and growth [3].

Exposure of peat to the atmosphere during dry periods enables more rapid decomposition of peat due to oxic conditions that enable aerobic respiration of soil organic matter. The frequency and duration of this exposure decrease with depth, so that gross peat loss declines with increasing water depth (Fig 2B):
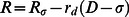
(4)where *R_σ_* is the rate of gross peat decomposition when water levels are at the optimum depth for sawgrass growth, and *r_d_* is the rate of respiration decline with depth. Like productivity in Eq. 3, respiration will vary over seasonal and inter-annual timescales, but Eq. 4 similarly represents the long-term mean behavior of gross peat decomposition as a function of depth, and thus integrates these faster dynamics. The simplifying assumption of linear changes in decomposition with depth must fail over some range of depths, but no empirical basis for a simple alternative functional form has been presented. In any case, the linear form is likely to approximate these relationships over the range of water levels relevant to ridge-slough formation, and model behavior will be comparable as long as the relationship is monotonic.

Combining Eqs. 1–4 and substituting 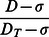 with 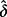 yields the more compact expression that describes reciprocal interactions between water depth and soil accrual:
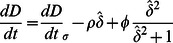
(5)Eq. 5 indicates that the local carbon balance model has only three independent parameters that govern its behavior: 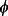, which is the difference between ridge and slough productivity 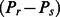; 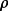, which is the respiration difference between the transition depth and sawgrass optimum 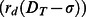, and 
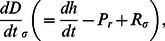
 which is the change in depth at the depth of maximum productivity.

Since equilibrium solutions to this point-scale model are both unwieldy and not particularly germane to our objectives, we simply note the qualitative behaviors that might emerge from these relationships. First, depending on the values of parameters, depth equilibria can occur at either shallow (ridges) or deeper (slough) depths or both (Fig. 2C–E). Several recent models of peat accretion responses to water-depth variation exhibit similar behaviors [11], [26], [27], [73], [74]. Second, the existence of depth equilibria and the peat accretion rates at those equilibria are dependent on which hydrologic parameters are fixed as boundary conditions. If water level is static (i.e., 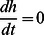), then these depth equilibria are also elevation equilibria, and the system will not accumulate soil. If 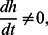 then depth equilibria are not elevation equilibria, and the landscape is stable when soil accretion or loss is equal to changes in the water level. The above behaviors will hold in situations where changes in soil elevation do not alter water elevation (i.e., water level is externally imposed by geologic features or active human management). However, in free-flowing systems such as the historic Everglades, flow and thus water depths would be relatively constant over time. In that case, water surface elevation is controlled by changes in soil elevation (i.e., a unit increase in soil elevation leads to a unit increase in water surface elevation), and the overlying water volume determines peat accretion since water depth would be insensitive to soil elevation change. The best available evidence suggests that the ridge-slough mosaic has been accreting peat since its establishment, while water depths of distinct ridges and sloughs remained relatively constant (as evinced by relatively stable plant communities [75]), the combination of which could not occur under any of the above assumptions. Thus a single-patch model of soil accretion in this landscape is insufficient to capture observed behaviors of the ridge-slough landscape.

### Lateral hydrologic coupling

**Figure 3:**
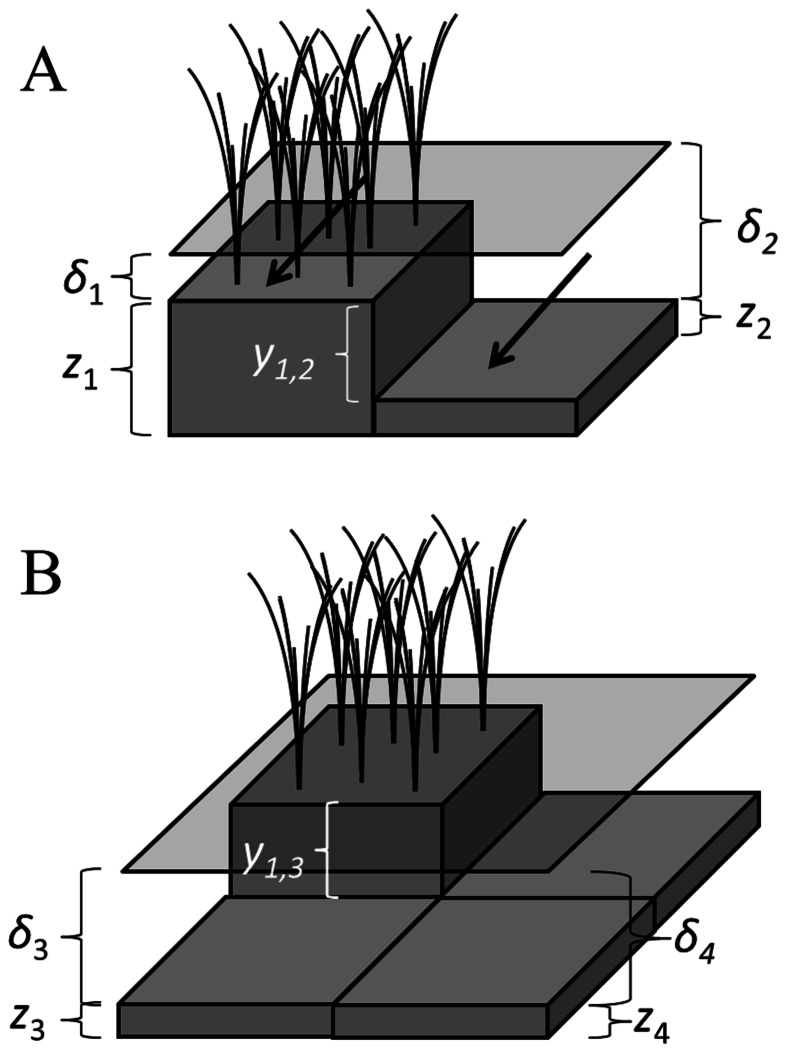
**Arrangement and description of patches in the (A) lateral and (B) longitudinal coupling models.** (a) Laterally-arrayed patches must convey discharge (Q) through their shared cross section, whose discharge capacity is driven by the sum of their depths (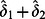). Stable landscapes occur when the difference in elevation between these patches (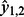) is at equilibrium (i.e., when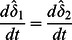). (b) For patches arrayed longitudinally, the higher (shallower) of each pair controls water levels. Stable landscapes occur when the differences in elevation between these patches 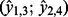 are at equilibrium (i.e., when 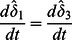 and 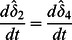). Lower elevation patches are shown as lacking vegetation, but actual sloughs contain dense mixtures of floating and submerged vegetation.



To assess feedbacks between carbon balance and discharge competence, we consider two patches of equal size, each with carbon balances that operate according to Eq. 5, arranged adjacently and perpendicular to the direction of hydrologic flow (Fig. 3A). Assuming sufficiently low gradient, water levels in the two patches will be equal, and changes in the soil elevation in either patch will influence that level, such that:

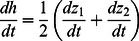
(6)Since peat production and decomposition are mediated by mean water depth, rather than elevation *per se*, our ultimate goal is to determine when relative and absolute depths of the coupled patches are at equilibrium (i.e., the landscape is stable). Combining Eqs. 2 and 6, we have that changes in depth in each of the two patches are:



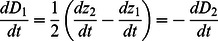
(7)Since 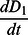 and 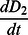 are equal in magnitude but opposite in sign, whenever one patch has a stable depth, the other patch does as well 
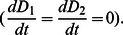
 Setting 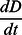 equal to zero in Eq. 5, substituting in for 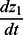 and 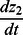 using Eq. 7, and combining terms gives:
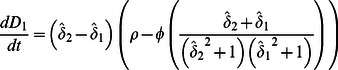
(8)The relationships described by Eq. 8 apply to situations where a fixed volume of water overlays soil patches. In order to incorporate the interactive effects of microtopography and flow on water level, we begin by defining *q_i_* as the specific discharge (i.e., discharge per unit width) entering the two patches from upstream and *v* as the depth-averaged velocity, and assume that each patch is of unit length. Water level for the two patches must satisfy:
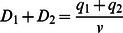
(9)We now define as the temporal mean of total discharge through the two patches, scaled to the difference between sawgrass optimum depth (σ) and transition depth (D_T_), and benchmarked against discharge at optimum sawgrass depth (σ × v):
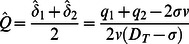
(10)For simplicity, we assume that velocity is constant with depth. Both modeling and empirical studies have shown modest changes in velocity with water depth variation in both ridges and sloughs [64], [76]–[78]; however, because of the low gradient and high roughness of the ridge-slough landscape, the majority of discharge variation is manifest as changes in depth rather than increased flow velocity [61]. As long as the sensitivity of velocity to water depth is small and monotonic, our assumption of constant velocity will have negligible influence on the behavior of this model.

A stable landscape in terms of vegetation and hydroperiod will occur when the relative depths of ridges and sloughs are constant. We therefore define 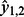 as the difference in elevation between the two patches, so that
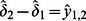
(11)Substituting Eq. 10 and 11 into Eq. 8 gives an equation in terms of total discharge conveyed by the two patches, their difference in elevation, and the carbon balance parameters *φ* and 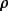:



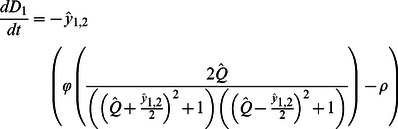
(12)Solutions to this equation in terms of 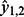 represent stable equilibria in elevation differences. Eq. 12 has one equilibrium at 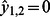 and four additional equilibria where elevations of the two patches are unequal, with their vertical separation given by:
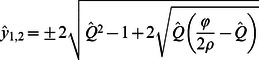
(13)or
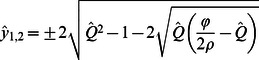
(14)The sign of 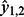 in Eqs. 13 and 14 dictates only whether 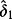 or 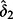 is greater, so we take the positive and negative cases to be equivalent solutions and consider only the negative case where 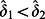 (i.e., 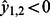 and thus 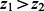).

The discharge range (if any) that supports divergent ridge and slough equilibria (i.e., where 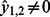 and thus 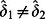) is controlled by the parameters *φ* and *ρ*, the values of which determine the relationship between soil accumulation and water depth (Fig. 2). For 
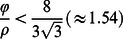
 real solutions to Eqs. 13 and 14 do not exist, and the solution at (i.e., a topographically homogenous landscape) is the only stable equilibrium for all ∧Q

**Figure 4:**
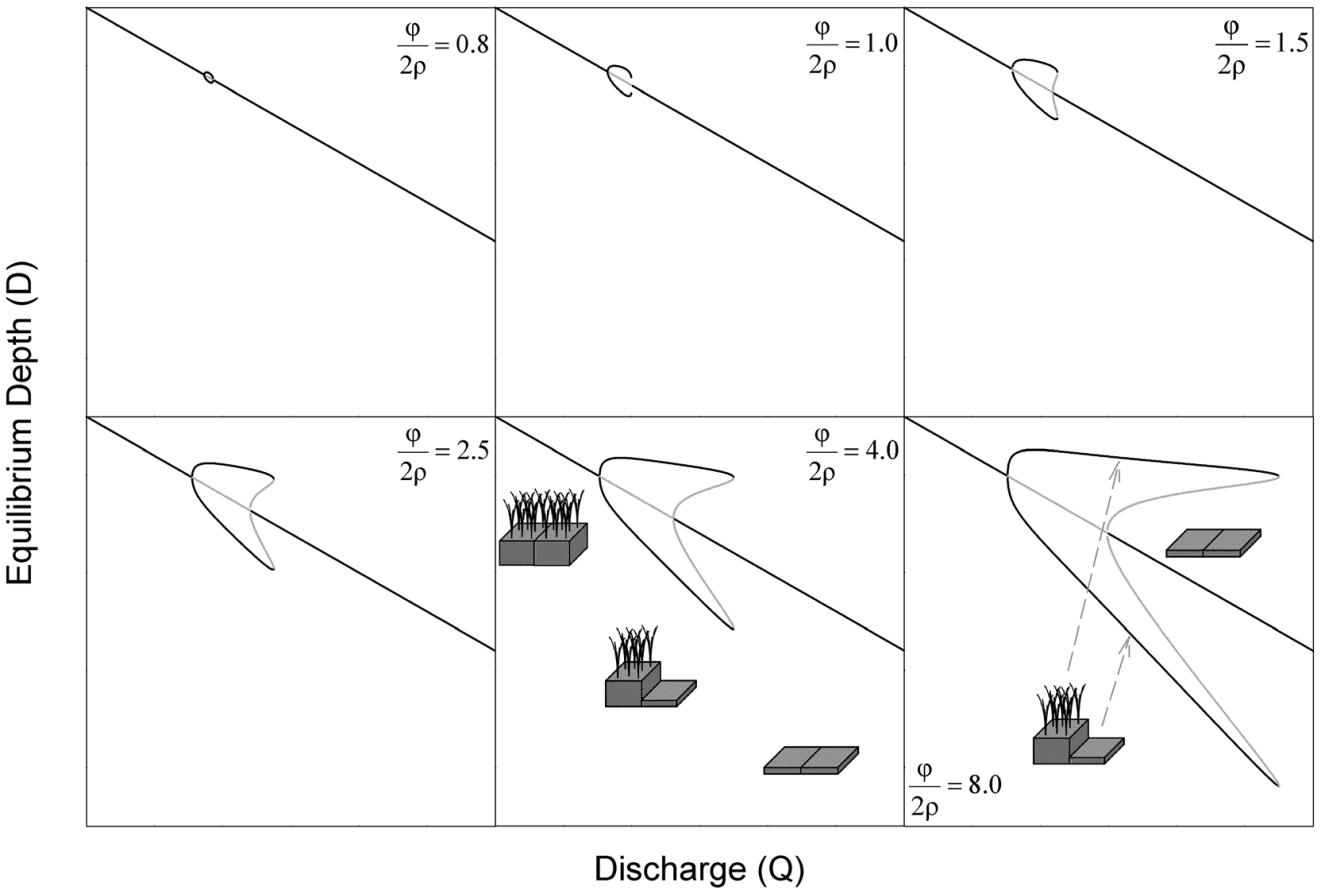
**Depth equilibria for the lateral coupling model as a function of discharge for different depth-accretion relationships (as described by the parameter ratio** 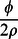 **; values are given in each panel).** ****Solid lines indicate stable equilibria, and dashed lines indicate unstable equilibria. (a) At low values of –, the discharge range that supports spontaneous divergence of ridges and sloughs is small, and homogenous landscape configuration is uniquely stable over large ranges of discharge. (b–f) The discharge range supporting spontaneous elevation divergence (i.e., where equal elevations are unstable) increases as differences in production between ridges and sloughs (φ) become larger or the slope of depth-respiration relationships (ρ) become smaller. If 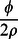 is sufficiently high (c–f), divergent elevations may exhibit bi-stability over a range of moderately high discharge, so that either the homogenous or divergent landscape is a stable equilibrium. The spontaneous divergence of elevations reflects the operation of distal negative feedbacks operating perpendicular to flow via discharge competence. Schematic symbols (from Fig. 2) indicate ranges of discharge that support divergent and homogenous elevation equilibria.

For 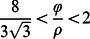, spontaneous divergence of ridges and sloughs occurs across a limited range of discharge, and either the homogenous or divergent landscape is uniquely stable. More specifically, the equilibrium described by Eq. 13 exists and is stable when 
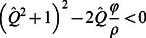
(15)but the equilibrium described by Eq. 14 has no real solutions. Within the range of discharge described by Eq. 15, the flat landscape is unstable, and adjacent patches will spontaneously diverge into distinct ridges and sloughs (Fig. 4a,b).

**Figure 5:**
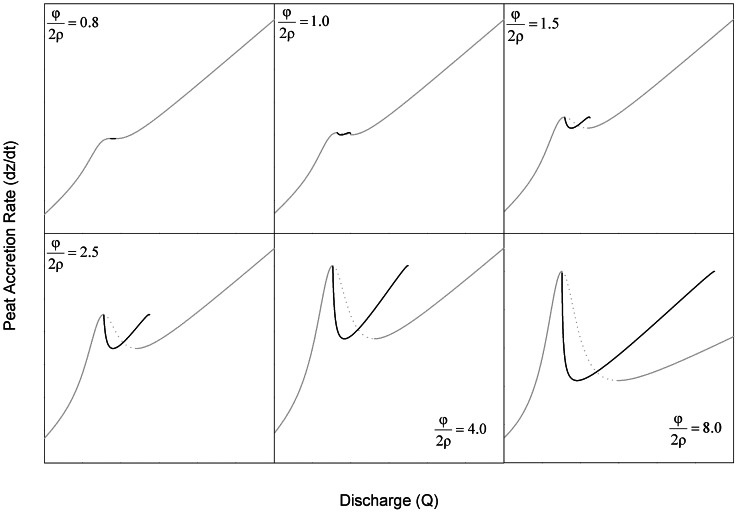
**Soil accretion as a function of discharge for different depth-accretion relationships, as indicated by the ratio of** 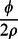. ****Grey lines indicate soil accretion rates for homogenous landcapes, and dashed grey lines indicate discharges at which homogenous landscapes are unstable. Black lines indicate soil accretion rates for heterogeneous landscapes (i.e., where unequal elevation of the two patches is a stable equilibrium). For the discharge range that supports spontaneous elevation divergence, soil accretion rates in hterogenous landscpes will be less than those predicted for a homogenous landscape. In the discharge region of bi-stability, heterogenous landscapes would be predicted to have greater soil accretion rates than homogenous landscapes. The magnitude of these disparities increases with the non-linearity of soil accretion-depth relationships (which increases with 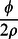).

Finally, for 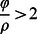, ridges and sloughs diverge spontaneously across the range of discharge described by Eq. 15. However, at still higher discharge, homogenous and divergent equilibria exhibit global bi-stability. The discharge bounds of the meta-stable region span from the upper bound of Eq. 15 to 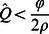. Within this range of discharge, the equilibrium given by Eq. 14 exists but is unstable, and sets the threshold separating the basins of attraction for the homogenous and divergent landscapes. The relative size of this region of bi-stability increases as soil accretion-water depth relationships become more non-linear (i.e., as 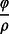 increases; Fig 4c-f). The parameters *φ* and *ρ* that determine the equilibrium difference in ridge slough elevation also influence the relationship between water depth or discharge and rates of soil accretion for the equilibrium landscape (Fig. 5). Regardless of parameter values, the overall trend is for peat accretion to increase with discharge; however, that relationship becomes markedly less linear and discontinuous as 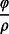 increases.

The effects of changes in discharge on soil accumulation are also dependent on topography. In particular, landscapes with divergent ridges and sloughs would be expected to have much steeper responses of soil accretion to discharge variation. Local maxima for peat accretion occur when ridge patches are near the optimum for sawgrass productivity; for topographically heterogeneous landscapes, peat accretion decreases sharply, and a second local maximum occurs at the greatest discharge that supports divergent ridges and sloughs. Within the discharge region that supports global bi-stability, soil accretion is predicted to be greater than in homogenous landscapes subject to the same hydrologic regime. Interestingly, this pattern suggests that bi-stability of this landscape arises specifically under discharge regimes where microtopographic heterogeneity promotes greater landscape-scale productivity than would occur under homogenous conditions, as has been suggested for arid-land systems [13], [19].

### Longitudinal hydrologic coupling – Model structure

For discharge competence-microtopography feedbacks to produce linear ridges and sloughs, the distal negative feedbacks generated must act more strongly parallel to flow than perpendicular to it. To evaluate the occurrence and strength of this potential anisotropy, we consider two additional patches (with elevation *z_3_* and *z_4_*) located immediately downstream (or upstream) of the laterally coupled patches (Fig. 3B). As in the lateral coupling model, accretion dynamics for all patches operate according to Eq. 12.

One assumption in this scenario is that the elevation of each downstream patch is equal to or lower than that of the patch directly upstream. This assumption implies that discharge capacity and thus water levels are controlled by the upstream patches, which can then be assumed to be at elevation equilibria (Fig. 4). In the case where one or both downstream patches are higher in elevation than their upstream counterparts, then downstream patches would control water level, and the upstream patches will behave according to the dynamics described below for *z*_3_ and *z*_4_.

Assuming that 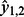 is at equilibrium (i.e., the depths of the upstream patches are stable) and discharge constant, then the change in water level is equal to the elevation change for the two upstream patches:
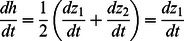
(16)


Next, we define 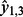 as the difference in elevation between the higher elevation upstream patch and its downstream neighbor 
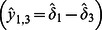
. Combining Eqs. 5 and 16 and substituting in 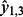, we see that a stable landscape (in terms of longitudinal elevation differences) occurs when:
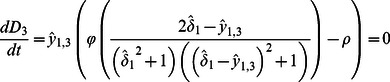
(17)


**Figure 6:**
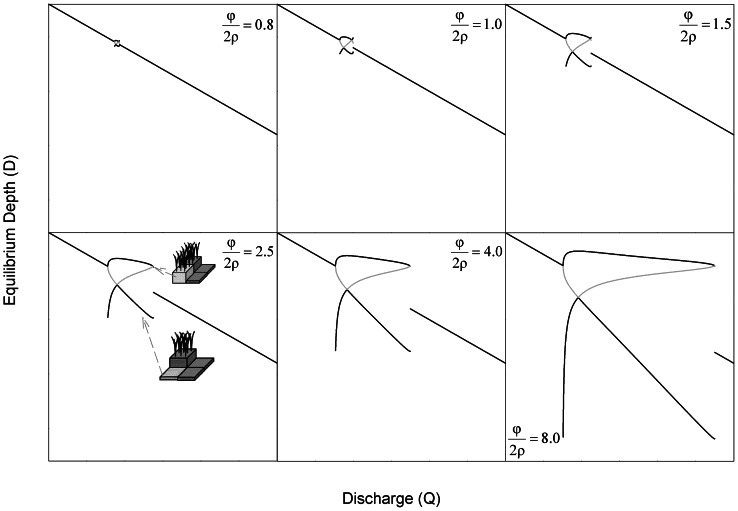
**Depth equilibria for the longitudinal coupling model as a function of discharge for different depth-accretion relationships.** All lines indicate depth equilibria for a patch whose water level is controlled by an upstream (or downstream) ridge patch at elevation equilibrium (Fig. 4). Black lines indicate stable equilibria, and grey lines indicate unstable equilibria. Under discharges that generate divergent elevation in laterally-connected patches, equal elevations of longitudinally-connected patches are always stable. For most such discharges, longitudinally-connected patches also have stable equilibria at which laterally (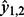) and longitudinally-connected (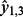) patches have equal depths (i.e. both are sloughs). Near thresholds separating divergent and homogenous landscapes, that coupling is relaxed, and downstream patches may have equilibria at slightly higher or much lower depths. However, downstream patches that become limiting to discharge capacity (i.e. shallower than upstream patches) are subject to the feedbacks and equilibria that constrain laterally-coupled patches (Fig. 4). The stability of equal elevations of longitudinally-arrayed patches reflects the weakness of flow-parallel discharge competence feedbacks in comparison to flow-perpendicular feedbacks. Schematic symbols (from Fig. 2) indicate ranges of discharge that support divergent and homogenous elevation equilibria.

The possible three equilibria occur either when upstream and downstream elevations are equal (i.e., when 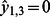 and thus 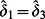); when the downstream patch has the same elevation as the upstream slough (i.e., 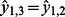 and thus 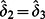); or where
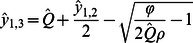
(18)Where divergent solutions for 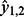 do not exist, the equilibrium at 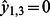 is the only stable solution. Since non-zero equilibria for 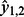 require that 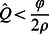, all solutions for 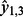 exist whenever laterally-adjacent patches have divergent equilibria. Across the range of 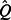 that supports the divergent equilibrium for 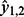, the order of relative elevations of solutions for 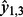 shift, but in all cases the intermediate elevation is unstable (Fig. 6). Over some range of 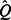, which decreases in size as – increases (Fig. 6), this transition results in one of the stable equilibria for 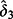 being less than the equilibrium for 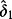. In that case, 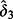 becomes limiting to discharge capacity, and, we assume, other elevations will adjust accordingly.

Throughout the discharge range that supports divergent topography, a patch located downstream of an established ridge will be stable as either a ridge or slough. The resilience of uniform longitudinal elevation, as measured by the relative size of its basin of attraction, is greatest at discharges near the center of the range that supports spontaneous divergence of laterally-coupled patches. At more extreme discharges, longitudinal divergence of ridges and sloughs becomes more likely as the basin of attraction becomes larger. One unexpected result of this analysis is that the equilibrium depth for sloughs that are downstream of ridges (Fig. 6) is predicted to increase sharply as discharge approaches the boundaries of the range supporting divergent topography. At high discharge, this equilibrium is equivalent to that for laterally-coupled sloughs (Fig. 4); however, at low discharge, the equilibrium elevation for sloughs downstream of ridges (Fig. 6) is much greater than for sloughs adjacent to ridges (Fig. 4).

Elevation equilibria for patches located downstream of sloughs are also described by Eq. 17 (substituting 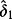 for 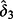, and 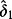 for 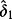), but our assumption that that downstream patches are lower in elevation dictates that the only viable configuration is where those patches are equal in elevation to upstream sloughs. More formally, for 
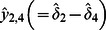
, the only stable equilibrium solution is 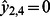. Other solutions to Eq. 17 require that 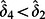, in which case 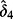 rather than 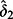 limits discharge competence and will therefore be coupled with the higher elevation patches (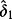 or 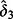) according to Eq. 12. Thus, any patch located downstream of a slough will also be a slough when our simplified model landscape is at an equilibrium.

The derivation and formal solutions for all equilibria and the bounds of their existence and stability are provided as Model Analysis S1. We confirmed analytical solutions numerically using spreadsheet calculations.

## Discussion

Our meta-ecosystem model of ecohydrologic interactions in wetlands relates the soil elevation of two patches that are hydrologically-connected (either laterally or longitudinally) but whose carbon balance (and thus soil accrual) operate locally. Analysis of this model suggests that feedbacks among soil elevation, peat production, and discharge competence have the potential to initiate formation of longitudinally-oriented patches of distinct elevation. Specifically, this model exhibits spontaneous divergence of adjacent soil elevations (i.e., formation of distinct ridges and sloughs) under some discharge conditions. This spontaneous divergence means that ridge establishment in one patch disfavors establishment in laterally-adjacent patches, and is thus locally self-reinforcing, but distally inhibitory.

In order to generate anisotropic pattern, the strength of this distally inhibitory feedback must depend on direction (Fig. 1D). Our analysis finds that in patches arranged longitudinally (parallel to flow), equal elevations always represent a stable (albeit not necessarily resilient) configuration, and divergence of ridges and sloughs never occurs spontaneously. Thus the distal negative feedback on peat topography generated by discharge competence operates more strongly in the flow-parallel than the flow perpendicular direction.

Our model presents a highly simplified representation of the ridge and slough landscape, in which directional interactions among patches are discrete and strong. In reality, the effects of topographic change at any given location on the broader landscape hydrologic regime are likely to be small, and their directionality far weaker than in our model. That is, the actual feedbacks among topography, soil accretion, and hydrologic conditions are far more diffuse than is represented by our model, with ecohydrologic feedbacks would be muted by the complex hydraulics of the ridge-slough landscape [76], [77]. As such, the context of patch transitions (between high-elevation ridges and low elevation sloughs) will determine the magnitude of their hydrologic effects. Because our model is not spatially-explicit, it cannot reproduce commonly observed phenomena, such as ridges that interrupt or bridge sloughs, which suggest relatively weak feedbacks (regardless of the spatial processes involved). Nonetheless, the directionality of feedbacks in our meta-ecosystem model may be sufficient to produce flow-parallel anisotropic pattern such as that observed in the Everglades, even in the absence of other spatially-dependent feedbacks such as sediment and/or nutrient redistribution.

A second limitation of this model is its focus on vertical differentiation, and consequent inability to predict specific length and width scales of ridges and sloughs. With sufficiently specified parameters for water depth – C balance relationships and rates of peat formation, our model could be used to predict elevation differences; conversely, elevation differences could be used to derive estimates of water depth – C balance relationships. The structure of our model does not allow for comparable estimates of length and width scales, which would require estimates of vegetation colonization rates and, potentially, gravitational and advective sediment movement to be implemented in a more spatially resolved model domain [26].

Finally, our model does not explicitly address the origin or role of tree islands within the ridge-slough landscape. Like ridges, tree islands are thought to be largely autogenic, and have degraded in response to hydrologic modification in some regions [41], [59]. Woody plants that occupy tree islands have particular hydrologic requirements, and tree establishment is likely to induce relatively discrete changes in productivity of recalcitrant organic matter (i.e. potential peat) [78]. Assuming that decomposition continues to increase monotonically with elevation, tree islands would reflect an additional local elevation equilibrium. Since inundation of tree islands was historically infrequent relative to ridges [41], flow occlusion by these patches would have effects on landscape discharge competence that are qualitatively to similar but larger than that of ridges [22]. Tree islands occupy only a small portion of even conserved landscapes, so their omission from this model is unlikely to qualitatively influence the results of our analysis. Nonetheless, more explicit integration of tree islands into models of ridge-slough landscape development and persistence are clearly needed.

Despite these limitations, the spontaneous vertical divergence of ridges and sloughs observed in our model is consistent with previous models of soil accretion dynamics in the Everglades and other peat systems [11], [27], [60], [73], [74]. However, this study is the first to demonstrate that discharge competence can generate directionally-dependent feedbacks that produce flow-parallel, anisotropic patterning. Some previous models addressing the spatial dynamics of peatlands have used one-dimensional approaches [27], [60], [79] to demonstrate the operation of distal feedbacks and the occurrence of multiple local attractors for peat accrual and elevation. Other models of peatland development have addressed the effects of nutrient redistribution and hydrologic regimes on the emergence of landscape pattern using two-dimensional landscapes [11], and demonstrated the potential for global bi-stability of landscape patterning [6], [79]. However, because those models address sub-surface rather than surface flow, hydrologic feedbacks have generated flow-perpendicular rather than flow-parallel patterning.

More recently, sediment redistribution via overland flow has been demonstrated as a plausible mechanism to generate a variety of landscape geometries, including linear, flow-oriented patches like those found in the Everglades ridge-slough landscape [25], [26]. However, the sediment redistribution mechanism in the Everglades requires sustained and regular periods during which the water slope diverges dramatically from the bed slope, events whose historical frequency is unclear. Moreover, the resulting model (RASCAL [25], [26]) does not address the potential effects of landscape configuration on hydroperiod under baseflow conditions [61]. The model presented here suggests that those effects may be sufficient to produce linear, flow-parallel landscape pattern.

### Assessing alternative patterning mechanisms

The proposed alternative mechanisms to explain the evolution of the ridge-slough landscape are, for the most part, mutually compatible and potentially self-reinforcing. All three mechanisms (sediment redistribution, nutrient accumulation, and discharge competence) rely on the occurrence of equal rates of land aggradation in ridges and sloughs to maintain stable relative elevations of landscape features. Moreover, none of the assumptions that underlie the hypotheses preclude the operation of the other processes. In fact, the operation of these and potentially other mechanisms seems likely, and their co-occurrence would make the emergence of linear patterning in the ridge-slough landscape more probable [39]. However, because the discharge competence hypothesis invokes the effects of landscape configuration on hydroperiod alone, while the sediment redistribution mechanism depends on regular occurrence of high velocities, the relative importance of these mechanisms has important implications for management of finite water volumes in the Everglades. As recently illustrated by Eppinga et al. [5], [12], [80], critical tests, in which the candidate mechanisms lead to divergent predictions, are needed to distinguish their relative importance and scales of operation.

The core assumptions that lead to spontaneous emergence of distinct ridges and sloughs in our model are generally supported by a few basic observations. First, since the ridge-slough landscape is a peat-accreting system, it is clear that positive carbon balances occur at relatively shallow depths favorable to sawgrass. Second, the much higher productivity of ridges than sloughs [68], [69] and their discrete distribution with depth supports the assumption of sharp transitions in productivity. Finally, the loss of peat following drainage of the Everglades (and other peatlands) makes clear that the relationship between water level and soil oxidation is negative [27]. Thus it is likely that values of *φ* and *ρ* in real peatland landscapes are within the range that could support spontaneous divergence of ridges and sloughs and other self-organized behavior in our model.

The creation of multiple attractors in local elevation via discharge competence (Fig. 4) is consistent with several observations about the historic and modern Everglades. Soil elevations are bi-modally distributed and anisotropic in ridge-slough regions where hydrologic modification has been least severe, and these sentinel attributes disappear under progressive drainage or impoundment [40]. In other words, there appear to be bounds on the occurrence of landscape patterning associated with water depth, consistent with the behavior of this model. This prediction is necessary to establish the plausibility of any proposed mechanism of ridge-slough formation, but since other models also predict the occurrence of hydrologic bounds to pattern emergence and persistence [26], discriminating among hypotheses on this basis may require more precise delineation of thresholds predicted by each mechanism.

The spontaneous divergence of ridge and slough elevations in our model is consistent with paleoecological studies that document the emergence of the ridge slough landscape approximately 1000–2000 years BP [75]. Available evidence suggests that sawgrass abundance increased dramatically under dryer-than-normal conditions at this time, emerging from a marsh-like landscape that supported the community of floating and emergent species now common to sloughs. The model presented in this paper predicts that spontaneous emergence of ridge-slough pattern would occur under relatively dry conditions (Fig. 4); in contrast, the sediment redistribution hypothesis would seemingly predict the emergence of landscape pattern under wetter conditions that would support prolonged high flow.

While the occurrence of distinct soil elevation modes is unlikely to discriminate among alternative mechanisms of ridge-slough formation, those mechanisms may predict distinct distributions of elevation among and within those modes. The discharge competence hypothesis makes a clear prediction that the elevation differences between ridges and sloughs should increase with increasing water depth or discharge (Fig. 4). Moreover, if depth-C-balance relationships are sufficiently non-linear (i.e., 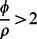), the discharge competence hypothesis would be supported by co-occurrence of bi-modal and unimodal elevation distributions at greater water depths. Finally, our model suggests that soil elevations might exhibit high variation and skewness near both the dry and wet boundaries of discharge that support ridge-slough persistence, in response to the deep elevation attractor in sloughs under dry conditions (Fig. 6).

Fine-scale features of both topographic and vegetative spatial pattern provide perhaps the most promising means of discriminating among alternative mechanisms of pattern formation. Models of sediment and nutrient re-distribution have both generated a variety of landscape geometries [11], [25], [55], but rarely have patterned wetlands been assessed for structure characteristic of self-organized patterning, including fractal patch geometry and distributions of (and relationships among) patch size and shape [81]–[83]. At present, the discharge competence has not been integrated with hydrodynamic landscape models that would allow comparable simulation of landscape dynamics, but this line of research is clearly a pressing need. Whether considering frequency distributions or spatial configuration, the core prediction of the discharge competence hypothesis is that the structure of microtopography will be best predicted by water depth and hydroperiod. In contrast, the sediment redistribution hypothesis would predict that those same metrics would be best predicted by spatial and temporal variation in flow velocity.

The discharge competence and sediment re-distribution hypotheses lead to distinct predictions about the temporal dynamics of topographic and vegetative responses to hydrologic change. According to the sediment re-distribution hypothesis, roughness resulting from vegetation structure, especially under transient conditions of high water slope, is an important influence on flow velocity and thus sediment redistribution [62], [63]. As such, changes in vegetation structure might be expected to precede changes in soil elevation distribution and patterning. In contrast, the discharge competence hypothesis invokes peat elevation as the primary control on hydrodynamics. Given potentially large C losses by drainage [41], peat elevations might be more responsive to hydrologic change than vegetation, particularly since the latter may exhibit priority affects. Limited evidence to date suggests that changes in vegetation composition and spatial structure lag behind changes in peat topography [40].

Finally, more robust empirical assessment of local C balance might allow both evaluation of core assumptions of the discharge competence hypothesis presented here and provide critical tests that would discriminate between the discharge competence and sediment redistribution hypotheses. First, relationships between depth and C fluxes would enable estimates of the critical parameters that govern topographic dynamics in our meta-ecosystem model; if discharge competence is the primary mechanism for ridge-slough development, then the ratio of these parameters will be large (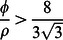; 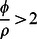 would provide stronger support). Moreover, the discharge competence hypothesis predicts that local C balance (GPP-ER) is equal and positive in both ridges and sloughs. In contrast, the sediment redistribution hypothesis is based on net transport of particulate organic matter from sloughs to ridges, in which case local net ecosystem production should be higher in the source (slough) habitat than in the sink (ridge).

### Implications for preservation and restoration of the ridge-slough landscape

Our meta-ecosystem model of the discharge competence hypothesis exhibits several behaviors with important implications for ecosystem restoration. The first of these is the possibility that the ridge-slough landscape could exhibit global bi-stability (i.e. that patterned and unpatterned landscapes represent alternative stable states). Alternative states are characterized by hysteretic responses to environmental drivers; in the Everglades, this would be manifest as pattern loss and re-establishment occurring at distinct hydrologic thresholds. As such, hydrologic conditions that enable persistence of landscape pattern may not suffice for its re-establishment, and degraded pattern may prove resistant to restoration. Even if hydrologic conditions are restored in the window for spontaneous re-establishment of ridge-slough formation, recovery is likely to be slow (but see [43]). On this basis, conservation of existing ridge-slough landscapes, rather than re-establishment of pattern in degraded portions of the greater Everglades, should perhaps be the higher priority [33].

Recent studies of critical transitions in self-organized systems have revealed spatial and temporal behaviors that may serve as leading indicators of pending regime shifts [84]. These indicators include changes in the structure of temporal variability [85], [86] and recovery from perturbation [87] as well as spatial variation [88], [89] and configuration [28], [90]. Unfortunately, indicators based on spatial pattern have proven less robust in landscapes with self-organized spatial structure [91], [92]. Our model suggests that the distribution of soil elevation within patch types, rather than at the scale of the entire landscape, may provide more robust predictors of pending pattern loss. Specifically, our analysis suggests that microtopographic change near critical discharge thresholds will be sensitive to the direction of hydrologic change. In landscapes subject to impoundment or increased flow volume, elevation of both ridges and sloughs will exhibit greater spatial variability (Fig. 4); in contrast, ridges should respond minimally to drainage, while slough elevations would be expected to exhibit a high degree of variability (Fig. 6). These results highlight important areas for future investigations of microtopographic structure as leading indicators of wetland pattern degradation. More generally, this study illustrates the value of simple models to assess spatial interactions and feedbacks and to generate diverse predictions against which hypothesized mechanisms can be tested.

## Supporting Information

Model Analysis S1 **Formal analysis of existence and stability of equilibria for lateral and longitudinal coupling models.**
(DOCX)
