# Supplementary material for: Discharge Competence and Pattern Formation in Peatlands: A Meta-Ecosystem Model of the Everglades Ridge-Slough Landscape
Source: PLoS One. 2013 May 9;8(5):e64174. doi: 10.1371/journal.pone.0064174 (PMC3650074; doi:10.1371/journal.pone.0064174)
Supplement: Model Analysis S1 — Formal analysis of existence and stability of equilibria for lateral and longitudinal coupling models. (DOCX) [file pone.0064174.s001.docx]

Heffernan, J. B., D. L. Watts, and M. J. Cohen. Discharge competence as an ecohydrologic mechanism for pattern formation in peatlands: a meta-ecosystem model of the Everglades ridge-slough landscape.

**Supporting Information: Model Analysis S1**

*Local C balance and elevation change:*

Water depth (D) is the difference between surface water level (h) and soil elevation (z):

$D=h-z$ (A1)

So that:

$\frac{dD}{dt}=\dot{D}=\frac{dh}{dt}-\frac{dz}{dt}=\dot{h}-\dot{z}$ (A2)

Local changes in soil elevation are driven by the balance of primary productivity (P) and decomposition (R):

$\frac{dz}{dt}=P-R$ (A3)

We model primary production with water depth as a function of water depth. In this model, gross primary production (P) has a maxima at the long-term mean water depth that is optimum for sawgrass growth (σ), and declines with increasing and decreasing depth:

$P=\left( P_{s}-P_{r} \right)\frac{\left( D-\sigma\right)^{2}}{\left( D-\sigma\right)^{2}+\left( D_{T}-\sigma\right)^{2}}+P_{r}$ (A4)

where *P*_s_ is the gross peat production in sloughs, *P*_r_ is gross peat production at the optimal depth for sawgrass growth, and *D_T_* is the depth at which peat accretion is the average of these two end-members (analogous to a half-saturation constant in monod kinetics).

Respiration declines with increasing water depth:

$R=R_{\sigma}-r_{d}\left( D-\sigma\right)$ (A5)

where *R_σ_* is the rate of gross peat decomposition when water levels are at the optimum depth for sawgrass growth, and r_d_ is the rate of respiration decline with depth.

We now define $\delta$ as water depth indexed to have a value of 0 when $D=\sigma$:

$\delta=h-z-\sigma$ (A6)

Substituting Eq. A6 in to expressions for productivity and respiration yields:

$P=\left( P_{s}-P_{r} \right)\frac{\delta^{2}}{\delta^{2}+\left( D_{T}-\sigma\right)^{2}}+P_{r}$ (A7)

and

$=R_{\sigma}-r_{D}\delta$ (A8)

Changes in elevation can therefore be described by:

$\frac{dz}{dt}=\left( P_{s}-P_{r} \right)\frac{\delta^{2}}{\delta^{2}+\left( D_{T}-\sigma\right)^{2}}+P_{r}-R_{\sigma}+r_{D}\left( \delta\right)$ (A9)

Let

$\delta=\hat{\delta}\cdot u_{D}=h-z-\sigma=D-\sigma$ (A10)

And

$\delta=\hat{\delta}\cdot u_{D}=\hat{\delta}\cdot\left( D_{T}-\sigma\right)$ (A11)

Substituting Eq. A11 into Eq. A9 yields

$\frac{dz}{dt}=\left( P_{s}-P_{r} \right)\frac{\hat{\delta}^{2}\left( D_{T}-\sigma\right)^{2}}{\hat{\delta}^{2}\left( D_{T}-\sigma\right)^{2}+\left( D_{T}-\sigma\right)^{2}}+P_{r}-R_{\sigma}+r_{D}\hat{\delta}\left( D_{T}-\sigma\right)$ (A12)

Which simplifies to:

$\frac{dz}{dt}=\left( P_{s}-P_{r} \right)\frac{\hat{\delta}^{2}}{\hat{\delta}^{2}+1}+P_{r}-R_{\sigma}+r_{D}\hat{\delta}\left( D_{T}-\sigma\right)$ (A13)

We now let

$\varphi=P_{r}-P_{s}$ (A14)

$\rho=r_{D}\left( D_{T}-\sigma\right)$ (A15)

So that Eq. A13 can be simplified to:

$\frac{dz}{dt}=P_{r}-R_{\sigma}+\rho\hat{\delta}-\varphi\frac{\hat{\delta}^{2}}{\hat{\delta}^{2}+1}$ (A16)

Substituting and simplifying yields

$\frac{dD}{dt}=\frac{dh}{dt}-\frac{dz}{dt}=\frac{dh}{dt}-P_{r}+R_{\sigma}-\rho\hat{\delta}+\varphi\frac{\hat{\delta}^{2}}{\hat{\delta}^{2}+1}$ (A17)

When $D=\sigma$, the change in elevation is

${\frac{dD}{dt}}_{\sigma} =\frac{dh}{dt}-P_{r}+R_{\sigma}$ (A18)

So that

$\frac{dD}{dt}={\frac{dD}{dt}}_{\sigma}-\rho\hat{\delta}+\varphi\frac{\hat{\delta}^{2}}{\hat{\delta}^{2}+1}$ (A19)

*Lateral coupling*

Local changes in depth resulting from peat accretion and changes in water level are described by:

$\frac{dD_{i}}{dt}={\frac{dD}{dt}}_{\sigma}-\rho\hat{\delta}_{i}+\varphi\frac{{\hat{\delta}_{i}}^{2}}{{\hat{\delta}_{i}}^{2}+1}$ (A20)

In our lateral coupling model, water must be routed through the shared cross section of the two adjacent patches, which therefore control water level:

$\frac{dh}{dt}=\frac{1}{2}\left( \frac{dz_{1}}{dt}+\frac{dz_{2}}{dt} \right)$ (A21)

Substituting in to Eq. A2 gives changes in depth for each patch in terms of changes in elevation of both patches:

$\frac{dD_{1}}{dt}=\frac{1}{2}\left( \frac{dz_{2}}{dt}+\frac{dz_{1}}{dt} \right)-\frac{dz_{1}}{dt}=\frac{1}{2}\left( \frac{dz_{2}}{dt}-\frac{dz_{1}}{dt} \right)$ (A22)

$\frac{dD_{2}}{dt}=\frac{1}{2}\left( \frac{dz_{2}}{dt}+\frac{dz_{1}}{dt} \right)-\frac{dz_{2}}{dt}=\frac{1}{2}\left( \frac{dz_{1}}{dt}-\frac{dz_{2}}{dt} \right)=-\frac{dD_{1}}{dt}$ (A23)

Any changes in depth in the two patches are thus equal in magnitude and opposite in sign. Combining Eqs. A20 and A23, we obtain:

$\frac{dD_{1}}{dt}=\frac{1}{2}\left( {\frac{dD}{dt}}_{\sigma}+\rho\hat{\delta}_{2}-\varphi\frac{{\hat{\delta}_{2}}^{2}}{{\hat{\delta}_{2}}^{2}+1}-{\frac{dD}{dt}}_{\sigma}-\rho\hat{\delta}_{1}+\varphi\frac{{\hat{\delta}_{1}}^{2}}{{\hat{\delta}_{1}}^{2}+1} \right)=0$ (A24)

Depth equilibria in the two patches therefore occur when:

$\frac{dD_{1}}{dt}=\frac{1}{2}\left( \rho\left( \hat{\delta}_{2}-\hat{\delta}_{1} \right)-\varphi\left( \frac{{\hat{\delta}_{2}}^{2}}{{\hat{\delta}_{2}}^{2}+1}-\frac{{\hat{\delta}_{1}}^{2}}{{\hat{\delta}_{1}}^{2}+1} \right) \right)=0$ (A25)

Which is true when:

$\left( \hat{\delta}_{2}-\hat{\delta}_{1} \right)\left( \rho-\varphi\left( \frac{\hat{\delta}_{1}+\hat{\delta}_{2}}{\left( {\hat{\delta}_{2}}^{2}+1 \right)\left( {\hat{\delta}_{1}}^{2}+1 \right)} \right) \right)=0$ (A26)

The depth in either patch is determined by discharge (*q*), which is a boundary condition, and velocity (*v*), which is assumed to be constant:

$D=\frac{q}{v}$ (A27)

The sum of discharges in the two patches is therefore:

$D_{1}+D_{2}=2\frac{q}{v}$ (A28)

When water depths in each patch are indexed to optimum depth for productivity (*σ*), Eq. A28 is equivalent to:

$D_{1}+D_{2}-2\sigma=2\frac{q}{v}-2\sigma$ (A29)

We now define$y_{1,2}$ as the difference in elevation between the two laterally-adjacent patches, so that:

$z_{2}=z_{1}+y_{1,2}$ (A30)

$D_{2}=D_{1}-y_{1,2}$ (A31)

$\delta_{2}=\delta_{1}-y_{1,2}$ (A32)

Combining Eq. A6 and A29, we have:

$\delta_{1}+\delta_{2}=2\frac{q}{v}-2\sigma$ (A33)

We now define

$\delta_{i}=\hat{\delta}_{1}\cdot u_{D}$ (A34)

So that:

$\hat{\delta}_{1}\cdot u_{D}+\hat{\delta}_{2}\cdot u_{D}=2\frac{q}{v}-2\sigma$ (A35)

From which it follows that:

$\hat{\delta}_{1}+\hat{\delta}_{2}=2\frac{q-\sigma v}{v\left( D_{T}-\sigma\right)}$ (A36)

Let $\hat{Q}$ be discharge, indexed to sawgrass optimum depth and scaled to $\left( D_{T}-\sigma\right):$

$\hat{Q}=\frac{q-\sigma v}{v\left( D_{T}-\sigma\right)}$ (A37)

It then follows from Eqs. A36 and A37 that:

$\hat{\delta}_{1}+\hat{\delta}_{2}=2\hat{Q}$ (A38)

We now define $\hat{y}$ as the difference in elevation so that:

$\hat{\delta}_{2}\cdot u_{D}=\hat{\delta}_{1}\cdot u_{D}-\hat{y}_{1,2}\cdot u_{D}$ (A39)

$\hat{\delta}_{2}=\hat{\delta}_{1}-\hat{y}_{1,2}$ (A40)

$\hat{\delta}_{1}-\hat{\delta}_{2}=\hat{y}_{1,2}$ (A41)

Substituting these identities into Eq. A26 yields:

$\hat{y}_{1,2}\left( \varphi\left( \frac{2\hat{Q}}{\left( {\hat{\delta}_{2}}^{2}+1 \right)\left( {\hat{\delta}_{1}}^{2}+1 \right)} \right)-\rho\right)=0$ (A42)

Combining Eqs. A38 and A41 yields:

$\hat{\delta}_{1}+\hat{\delta}_{1}-\hat{y}_{1,2}=2Q$ (A43)

From which we have that:

$\hat{\delta}_{1}=\hat{Q}+\frac{\hat{y}_{1,2}}{2}$ (A44)

$\hat{\delta}_{2}=\hat{Q}-\frac{\hat{y}_{1,2}}{2}$ (A45)

We now substitute Eqs. A44 and A45 into Eq. A42 to obtain the final form of the equation describing changes in depth:

$\frac{dD_{1}}{dt}=\hat{y}_{1,2}\left( \varphi\left( \frac{2\hat{Q}}{\left( \left( \hat{Q}+\frac{\hat{y}_{1,2}}{2} \right)^{2}+1 \right)\left( \left( \hat{Q}-\frac{\hat{y}_{1,2}}{2} \right)^{2}+1 \right)} \right)-\rho\right)$ (A46)

*Equilibrium solutions for lateral coupling model*

Depth equilibria occur when

$\frac{dD_{1}}{dt}=0$ (A47)

Which is equivalent to:

$\hat{y}_{1,2}\left( \varphi\left( \frac{2\hat{Q}}{\left( \left( \hat{Q}+\frac{\hat{y}_{1,2}}{2} \right)^{2}+1 \right)\left( \left( \hat{Q}-\frac{\hat{y}_{1,2}}{2} \right)^{2}+1 \right)} \right)-\rho\right)=0$ (A48)

Eq. A48 is true when:

$\hat{y}_{1,2}=0$ (A49)

Or

$\varphi\left( \frac{2\hat{Q}}{\left( \frac{\hat{y}_{1,2}}{2} \right)^{4}+2\left( 1-\hat{Q}^{2} \right)\left( \frac{\hat{y}_{1,2}}{2} \right)^{2}+\left( 1+\hat{Q}^{2} \right)^{2}} \right)-\rho=0$ (A50)

Eq. A50 may be expressed as a quadratic in $\left( \frac{\hat{y}_{1,2}}{2} \right)^{2}$:

$\left( \frac{\hat{y}_{1,2}}{2} \right)^{4}+2\left( 1-\hat{Q}^{2} \right)\left( \frac{\hat{y}_{1,2}}{2} \right)^{2}+\left( 1+\hat{Q}^{2} \right)^{2}-2\hat{Q}\left( \frac{\varphi}{\rho} \right)=0$ (A51)

Which has solutions:

$\left( \frac{\hat{y}_{1,2}}{2} \right)^{2}=\frac{-b\pm\sqrt{b^{2}-4ac}}{2a}$ (A52)

where

$a=1$ (A53)

$b=2\left( 1-\hat{Q}^{2} \right)$ (A54)

$c=\left( 1+\hat{Q}^{2} \right)^{2}-2\hat{Q}\left( \frac{\varphi}{\rho} \right)$ (A55)

Since

$b^{2}=4\left( 1-\hat{Q}^{2} \right)^{2}$ (A56)

and

$-4ac=8\hat{Q}\left( \frac{\varphi}{\rho} \right)-4\left( 1+\hat{Q}^{2} \right)^{2}$ (A57)

It follows that:

$b^{2}-4ac=4\left( 1-\hat{Q}^{2} \right)^{2}+8\hat{Q}\left( \frac{\varphi}{\rho} \right)-4\left( 1+\hat{Q}^{2} \right)^{2}=$ (A58)

Which can simplified to:

$b^{2}-4ac=8\hat{Q}\left( \frac{\varphi}{\rho} \right)-16\hat{Q}^{2}=$ (A59)

$b^{2}-4ac=16\hat{Q}\left( \frac{\varphi}{2\rho}-\hat{Q} \right)$ (A60)

Therefore solutions to Eq. A48 are:

$\left( \frac{\hat{y}_{1,2}}{2} \right)^{2}=\frac{-2\left( 1-\hat{Q}^{2} \right)\pm4\sqrt{\hat{Q}\left( \frac{\varphi}{2\rho}-\hat{Q} \right)}}{2}$ (A61)

Since the designation of patches 1 and 2 are entirely arbitrary, we take the positive and negative roots for values of $\hat{y}_{1,2}$ solutions to be equivalent solutions. Equilibrium depths for laterally-adjacent patches therefore occur when:

$\hat{y}_{1,2}=-2\sqrt{\hat{Q}^{2}-1\pm2\sqrt{\hat{Q}\left( \frac{\varphi}{2\rho}-\hat{Q} \right)}}$ (A62)

*Bounding existence of solutions to lateral coupling model*

The trivial solution to Eq. A46 at $\hat{y}_{1,2}=0$ always exists.

Solutions at

$\hat{y}_{1,2}=-2\sqrt{\hat{Q}^{2}-1\pm2\sqrt{\hat{Q}\left( \frac{\varphi}{2\rho}-\hat{Q} \right)}}$ (A63)

Exist only if

$\hat{Q}\left( \frac{\varphi}{2\rho}-\hat{Q} \right)>0$ (A64)

Which requires

$0<\hat{Q}<\frac{\varphi}{2\rho}$ (A65)

Assuming $\frac{\varphi}{2\rho}>0$, there is always some Q for which this is true.

The solution at

$\hat{y}_{1,2}=-2\sqrt{\hat{Q}^{2}-1+2\sqrt{\hat{Q}\left( \frac{\varphi}{2\rho}-\hat{Q} \right)}}$ (A66)

Further requires that

$\hat{Q}^{2}-1+2\sqrt{\hat{Q}\left( \frac{\varphi}{2\rho}-\hat{Q} \right)}>0$ (A67)

$1-\hat{Q}^{2}<2\sqrt{\hat{Q}\left( \frac{\varphi}{2\rho}-\hat{Q} \right)}$ (A68)

This is true for $1<\hat{Q}<\frac{\varphi}{2\rho}$, over which range a real solution to Eq. A66 always exists.

For $\hat{Q}<1$, a real solution to Eq. A66 exists when:

$1-\hat{Q}^{2}<2\sqrt{\hat{Q}\left( \frac{\varphi}{2\rho}-\hat{Q} \right)}$ (A69)

Which is equivalent to:

$\hat{Q}^{4}+2\hat{Q}^{2}-2\hat{Q}\frac{\varphi}{\rho}+1<0$ (A70)

Since the value of Eq. A70 at the limit is positive, a real solution only exists for $\hat{Q}<1$ if

$\hat{Q}^{4}+2\hat{Q}^{2}-2\hat{Q}\frac{\varphi}{\rho}+1=0$ (A71)

Has real solutions. The discriminant for a quartic function ($D_{4})$is:

$$D_{4}=\left( a_{1}^{2}a_{2}^{2}a_{3}^{2}-4a_{1}^{3}a_{3}^{3}-4a_{1}^{2}a_{2}^{3}a_{4}+18a_{1}^{3}a_{2}a_{3}a_{4}-27a_{1}^{4}a_{4}^{2}+256a_{0}^{3}a_{4}^{3} \right)+a_{0}\left( -4a_{2}^{3}a_{3}^{2}+18a_{1}a_{2}a_{3}^{3}+16a_{2}^{4}a_{4}-80a_{1}a_{2}^{2}a_{3}a_{4}-6a_{1}^{2}a_{3}^{2}a_{4}+144a_{1}^{2}a_{2}a_{4}^{2} \right)+a_{0}^{2}\left( -27a_{3}^{4}+144a_{2}a_{3}^{2}a_{4}-128a_{2}^{2}a_{4}^{2}-192a_{1}a_{3}a_{4}^{2} \right)$$

(A72)

Eq. A71 has real solutions if $D_{4}<0$. For Eq. A71, parameters of the discriminant are:

$a_{0}=1$ (A73)

$a_{1}=-2\frac{\varphi}{\rho}$ (A74)

$a_{2}=2$ (A75)

$a_{3}=0$ (A76)

$a_{4}=1$ (A77)

Substituting these parameters in to the generic determinant formula yields:

$D_{4}=-32a_{1}^{2}-27a_{1}^{4}+256+256+288a_{1}^{2}-512$ (A78)

Which simplifies to:

$D_{4}=256a_{1}^{2}-27a_{1}^{4}$ (A79)

$D_{4}=a_{1}^{2}\left( 256-108\left( \frac{\varphi}{\rho} \right)^{2} \right)$ (A80)

Eq. A71 has real solutions when:

$a_{1}^{2}\left( 256-108\left( \frac{\varphi}{\rho} \right)^{2} \right)<0$ (A81)

Which is true only when:

$\frac{\varphi}{\rho}>\frac{8}{3\sqrt{3}}$ (A82)

The actual value of the solutions to Eq. A70, which determine the discharge at which bifurcations occur, are given by Eqs. A110-112. We present analyses of these solutions after some preliminary analysis of the other nonzero solution.

The solution at

$\hat{y}_{1,2}=-2\sqrt{\hat{Q}^{2}-1-2\sqrt{\hat{Q}\left( \frac{\varphi}{2\rho}-\hat{Q} \right)}}$ (A83)

Is real if

$\hat{Q}^{2}-1>2\sqrt{\hat{Q}\left( \frac{\varphi}{2\rho}-\hat{Q} \right)}$ (A84)

Which is never true for $\hat{Q}<1$. Since $0<\hat{Q}<\frac{\varphi}{2\rho}$, it follows that a real solution exists when $\frac{\varphi}{\rho}>2$.

Assuming $\hat{Q}>1$, Eq. A83 has a real solution when

$\hat{Q}^{2}-1>2\sqrt{\hat{Q}\left( \frac{\varphi}{2\rho}-\hat{Q} \right)}$ (A85)

Which is equivalent to:

$\hat{Q}^{4}+2\hat{Q}^{2}-2\hat{Q}\frac{\varphi}{\rho}+1>0$ (A86)

Given the generic quartic equation:

$Ax^{4}+Bx^{3}+Cx^{2}+Dx+E$ (A87)

its solution can be found by means of the following calculations (Cardano’s solution). Let:

$\alpha=-\frac{3B^{2}}{8A^{2}}+\frac{C}{A}$ (A88)

$\beta=\frac{B^{3}}{8A^{3}}-\frac{BC}{2A^{2}}+\frac{D}{A}$ (A89)

$\gamma=-\frac{3B^{4}}{256A^{4}}+\frac{CB^{2}}{16A^{3}}-\frac{BD}{24}+\frac{E}{A}$ (A90)

For Eq. A86,

$\alpha=2$ (A91)

$\beta=-\frac{2\varphi}{\rho}$ (A92)

$\gamma=1$ (A93)

We now let:

$P=-\frac{\alpha^{2}}{12}-\gamma$ (A94)

$Q=-\frac{\alpha^{3}}{108}+\frac{\alpha\gamma}{3}-\frac{\beta^{3}}{8}$ (A95)

$R=-\frac{Q}{2}\pm\sqrt{\frac{Q^{2}}{4}+\frac{P^{3}}{27}}$ (A96)

For Eq. A86,

$Q=-\frac{8}{108}+\frac{2}{3}-\frac{1}{2}\left( \frac{\varphi}{\rho} \right)^{2}=\frac{16}{27}-\frac{1}{2}\left( \frac{\varphi}{\rho} \right)^{2}$ (A97)

$P=-\frac{4}{12}-1=-\frac{4}{3}$ (A98)

$R=\left( \frac{\varphi}{2\rho} \right)^{2}-\frac{8}{27}\pm\left( \frac{\varphi}{2\rho} \right)\sqrt{\left( \frac{\varphi}{2\rho} \right)^{2}-\frac{16}{27}}$ (A99)

We now let

$U=\sqrt[3]{R}=\sqrt[3]{\left( \frac{\varphi}{2\rho} \right)^{2}-\frac{8}{27}+\left( \frac{\varphi}{2\rho} \right)\sqrt{\left( \frac{\varphi}{2\rho} \right)^{2}-\frac{16}{27}}}$ (A100)

And let

$y=-\frac{5}{6}\alpha+U-\frac{P}{3U}=-\frac{5}{3}+U+\frac{4}{9U}$ (A101)

So that

$y=\frac{9U^{2}-15U+4}{9U}$ (A102)

We further let

$W=\sqrt{\alpha+2y}$ (A103)

So that

$W=\sqrt{2+2\left( -\frac{5}{3}+U+\frac{4}{9U} \right)}=\sqrt{-\frac{4}{3}+2U+\frac{8}{9U}}=\sqrt{\frac{18U^{2}-12U+8}{9U}}$ (A104)

Solutions to the quartic equation are given by:

$x=-\frac{B}{4a}+\frac{\pm_{s} W\mp_{t}\sqrt{-(3\alpha+2y\pm\frac{2\beta}{W}}}{2}$ (A105)

For Eq. A86, the root

$\sqrt{-(3\alpha+2y\pm\frac{2\beta}{W}}$ (A106)

Is equivalent to

$\sqrt{-\left( 6+2\left( -\frac{5}{3}+U+\frac{4}{9U} \right)+\frac{2\beta}{\sqrt{\frac{18U^{2}-12U+8}{9U}}} \right)}=\sqrt{-\left( 3\alpha-\frac{5}{3}+2U-\frac{8}{9U}+\frac{2\beta}{W} \right)}$ (A107)

$=\sqrt{-\left( \frac{4}{3}\alpha+2U-\frac{2P}{3U}+\frac{2\beta}{W} \right)}$ (A108)

$=\sqrt{4\frac{\varphi}{\rho}\frac{1}{W}-4-W^{2}}$ (A109)

Solutions to Eq. A86 occur at

$Q=\frac{W}{2}\pm\sqrt{4\frac{\varphi}{\rho}\frac{1}{W}-4-W^{2}}$ (A110)

Where

$W=\sqrt{\frac{18U^{2}-12U+8}{9U}}$ (A111)

And

$U=\sqrt[3]{\left( \frac{\varphi}{2\rho} \right)^{2}-\frac{8}{27}+\left( \frac{\varphi}{2\rho} \right)\sqrt{\left( \frac{\varphi}{2\rho} \right)^{2}-\frac{16}{27}}}$ (A112)

**Summary of bifurcations**

The upper bound of both equilibria is $\hat{Q}<\frac{\varphi}{2\rho}$.

The lower bound of the equilibrium depth difference

$\hat{y}_{1,2}=-2\sqrt{\hat{Q}^{2}-1-2\sqrt{\hat{Q}\left( \frac{\varphi}{2\rho}-\hat{Q} \right)}}$ (A113)

Requires $\hat{Q}>1$, and so occurs when

$Q=\frac{W}{2}+\sqrt{4\frac{\varphi}{\rho}\frac{1}{W}-4-W^{2}}$ (A114)

This solution exists only if $\frac{\varphi}{\rho}>2.$

The lower bound of the equilibrium depth difference

$\hat{y}_{1,2}=-2\sqrt{\hat{Q}^{2}-1+2\sqrt{\hat{Q}\left( \frac{\varphi}{2\rho}-\hat{Q} \right)}}$ (A115)

Requires $\hat{Q}<1$, and so occurs when

$Q=\frac{W}{2}-\sqrt{4\frac{\varphi}{\rho}\frac{1}{W}-4-W^{2}}$ (A116)

This solution exists only when $\frac{\varphi}{\rho}>\frac{8}{3\sqrt{3}}$

*Stability of solutions to lateral coupling model*

To assess the stability of equilibrium depth differences, we take the derivative of Eq. A46 with respect to $\hat{y}_{1,2}$, which is:

$\frac{d\dot{D}_{1}}{d\hat{y}_{1,2}}=\frac{2\varphi\hat{Q}\left( -3\left( \frac{\hat{y}_{1,2}}{2} \right)^{4}-3\left( 1-\hat{Q}^{2} \right)\left( \frac{\hat{y}_{1,2}}{2} \right)^{2}+\left( 1+\hat{Q}^{2} \right)^{2} \right)}{\left( \left( \frac{\hat{y}_{1,2}}{2} \right)^{4}+2\left( 1-\hat{Q}^{2} \right)\left( \frac{\hat{y}_{1,2}}{2} \right)^{2}+\left( 1+\hat{Q}^{2} \right)^{2} \right)^{2}}-\rho$ (A117)

Evaluating this derivative for $\hat{y}_{1,2}=0$ yields:

${\frac{d\dot{D}_{1}}{d\hat{y}_{1,2}}}_{y1,2=0}=\frac{2\varphi\hat{Q}}{\left( 1+\hat{Q}^{2} \right)^{2}}-\rho$ (A118)

The equilibrium is stable when

$\frac{2\varphi\hat{Q}}{\left( 1+\hat{Q}^{2} \right)^{2}}-\rho<0$ (A119)

Which is equivalent to

$\left( 1+\hat{Q}^{2} \right)^{2}-2\hat{Q}\frac{\varphi}{\rho}>0$ (A120)

So the equilibrium at $\hat{y}_{1,2}=0$ is unstable between the lower bounds (bifurcations) for the other two equilibria.

The derivative of Eq. A46 can also be expressed as:

$\frac{d\dot{D}_{1}}{d\hat{y}_{1,2}}=\frac{\left( \left( \frac{\hat{y}_{1,2}}{2} \right)^{4}+2\left( 1-\hat{Q}^{2} \right)\left( \frac{\hat{y}_{1,2}}{2} \right)^{2}+\left( 1+\hat{Q}^{2} \right)^{2} \right)2\varphi\hat{Q}-2\varphi\hat{Q}\hat{y}_{1,2}\left( 2\left( \frac{\hat{y}_{1,2}}{2} \right)^{3}+\left( 1-\hat{Q}^{2} \right)\hat{y}_{1,2} \right)}{\left( \left( \frac{\hat{y}_{1,2}}{2} \right)^{4}+2\left( 1-\hat{Q}^{2} \right)\left( \frac{\hat{y}_{1,2}}{2} \right)^{2}+\left( 1+\hat{Q}^{2} \right)^{2} \right)^{2}}-\rho$ (A121)

Eq. A51 can be re-arranged as:

$\left( \frac{\hat{y}_{1,2}}{2} \right)^{4}+2\left( 1-\hat{Q}^{2} \right)\left( \frac{\hat{y}_{1,2}}{2} \right)^{2}+\left( 1+\hat{Q}^{2} \right)^{2}=2\hat{Q}\left( \frac{\varphi}{\rho} \right)$ (A122)

Which we substitute in to Eq. A121:

$\frac{d\dot{D}_{1}}{d\hat{y}_{1,2}}=\frac{\left( \frac{2\varphi\hat{Q}}{\rho} \right)2\varphi\hat{Q}-2\varphi\hat{Q}\hat{y}_{1,2}\left( 2\left( \frac{\hat{y}_{1,2}}{2} \right)^{3}+\left( 1-\hat{Q}^{2} \right)\hat{y}_{1,2} \right)}{\left( \frac{2\varphi\hat{Q}}{\rho} \right)^{2}}-\rho$ (A123)

This expression simplifies to:

$\frac{d\dot{D}_{1}}{d\hat{y}_{1,2}}=-\frac{\rho^{2}\left( \hat{y}_{1,2} \right)^{2}\left( \left( \frac{\hat{y}_{1,2}}{2} \right)^{2}+\left( 1-\hat{Q}^{2} \right) \right)}{2\varphi\hat{Q}}$ (A124)

Evaluating for the equilibrium given by Eq. A62, we have that

$\left( \frac{\hat{y}_{1,2}}{2} \right)^{2}=\hat{Q}^{2}-1+2\sqrt{\hat{Q}\left( \frac{\varphi}{2\rho}-\hat{Q} \right)}$ (A125)

Which we substitute into Eq. A124 to obtain:

$\frac{d\dot{D}_{1}}{d\hat{y}_{1,2}}=-\frac{\rho^{2}\left( \hat{y}_{1,2} \right)^{2}\left( 2\sqrt{\hat{Q}\left( \frac{\varphi}{2\rho}-\hat{Q} \right)} \right)}{2\varphi\hat{Q}}$ (A126)

Since both the numerator and the denominator are always positive, the equilibrium at

$\hat{y}_{1,2}=-2\sqrt{\hat{Q}^{2}-1+2\sqrt{\hat{Q}\left( \frac{\varphi}{2\rho}-\hat{Q} \right)}}$ (A127)

Is always stable when it exists.

Evaluating for the equilibrium given by Eq. A62, we have that

$\left( \frac{\hat{y}_{1,2}}{2} \right)^{2}=\hat{Q}^{2}-1-2\sqrt{\hat{Q}\left( \frac{\varphi}{2\rho}-\hat{Q} \right)}$ (A128)

Which we substitute into Eq. A124 to obtain:

$\frac{d\dot{D}_{1}}{d\hat{y}_{1,2}}=\frac{\rho^{2}\left( \hat{y}_{1,2} \right)^{2}\left( 2\sqrt{\hat{Q}\left( \frac{\varphi}{2\rho}-\hat{Q} \right)} \right)}{2\varphi\hat{Q}}$ (A129)

Since the numerator and denominator are always positive, the equilibrium at

$\hat{y}_{1,2}=-2\sqrt{\hat{Q}^{2}-1-2\sqrt{\hat{Q}\left( \frac{\varphi}{2\rho}-\hat{Q} \right)}}$ (A130)

Is always unstable when it exists.

*Longitudinal coupling*

We now consider a third patch, with soil elevaton $z_{3}$, located downstream of the deeper of two patches adjacent upstream patches (i.e. $D_{1}$ and $D_{2})$. Further, we assume that $D_{1}$ and $D_{2}$ at equilibrium, meaning that

$\hat{y}_{1,2}=-2\sqrt{\hat{Q}^{2}-1+2\sqrt{\hat{Q}\left( \frac{\varphi}{2\rho}-\hat{Q} \right)}}$ (A131)

or

$\hat{y}_{1,2}=0$ (A132)

Since the equilibrium at

$\hat{y}_{1,2}=-2\sqrt{\hat{Q}^{2}-1-2\sqrt{\hat{Q}\left( \frac{\varphi}{2\rho}-\hat{Q} \right)}}$ (A133)

Is unstable and so unlikely to persist as a constraint on water levels to patch $D_{3}$. We take as given that $D_{1}$is the higher elevation (shallower depth) of the two upstream patches.

Changes in depth are governed by the same carbon balance responses to water depth as the upstream patches

$\frac{{dD}_{3}}{dt}=\frac{dh}{dt}-\frac{dz_{3}}{dt}=\frac{dh}{dt}-\dot{z}_{0}+\rho\hat{\delta}_{3}-\varphi\frac{{\hat{\delta}_{3}}^{2}}{{\hat{\delta}_{3}}^{2}+1}$ (A134)

However, we assume that $z_{3}\leq z_{1}$ so that $z_{3}$does not control but only responds to water levels.

Changes in water level for the downstream patch ($\hat{\delta}_{3}$) are dependent on the changes in elevation (and thus water level) of the two upstream patches:

$\frac{dh}{dt}=\frac{1}{2}\left( \frac{dz_{1}}{dt}+\frac{dz_{2}}{dt} \right)=\frac{dz_{1}}{dt}$ (A135)

So that changes in depth for the downstream patch are governed by:

$\frac{{dD}_{3}}{dt}={\frac{dD}{dt}}_{\sigma}-\rho\hat{\delta}_{1}+\varphi\frac{{\hat{\delta}_{1}}^{2}}{{\hat{\delta}_{1}}^{2}+1}-{\frac{dD}{dt}}_{\sigma}+\rho\hat{\delta}_{3}-\varphi\frac{{\hat{\delta}_{3}}^{2}}{{\hat{\delta}_{3}}^{2}+1}$ (A136)

Letting

$z_{3}=z_{1}+y_{1,3}$ (A137)

$D_{3}=D_{1}-y_{1,3}$ (A138)

$\delta_{3}=\delta_{1}-y_{1,3}$ (A139)

$\hat{y}_{1,3}=\hat{\delta}_{1}-\hat{\delta}_{3}$ (A140)

Eq. A136 can be simplified to:

$\frac{{dD}_{3}}{dt}=\hat{y}_{1,3}\left( \varphi\frac{2\hat{\delta}_{1}-\hat{y}_{1,3}}{\left( {\hat{\delta}_{1}}^{2}+1 \right)\left( \left( \hat{\delta}_{1}-\hat{y}_{1,3} \right)^{2}+1 \right)}-\rho\right)$ (A141)

Equilibria occur where

$\hat{y}_{1,3}\left( \varphi\frac{2\hat{\delta}_{1}-\hat{y}_{1,3}}{\left( {\hat{\delta}_{1}}^{2}+1 \right)\left( \left( \hat{\delta}_{1}-\hat{y}_{1,3} \right)^{2}+1 \right)}-\rho\right)=0$ (A142)

The trivial solution ($\hat{y}_{1,3}=0)$ is equivalent to:

$\therefore\hat{\delta}_{3}=\hat{\delta}_{1}$ (A143)

Additional solutions occur when:

$\varphi\frac{2\hat{\delta}_{1}-\hat{y}_{1,3}}{\left( {\hat{\delta}_{1}}^{2}+1 \right)\left( \left( \hat{\delta}_{1}-\hat{y}_{1,3} \right)^{2}+1 \right)}-\rho=0$ (A144)

Since $D_{3}$ follows same rules as $D_{1}$, it seems intuitive that an equilibrium would also occur when

$\therefore\hat{\delta}_{3}=\hat{\delta}_{2}$ (A145)

From which it follows that

$\hat{\delta}_{3}=\hat{\delta}_{1}-\hat{y}_{1,3}=\hat{\delta}_{2}=\hat{\delta}_{1}-\hat{y}_{1,2}$ (A146)

and

$\hat{y}_{1,3}=\hat{y}_{1,2}$ (A147)

So that Eq. A141 would be equivalent to:

$\varphi\frac{2\hat{Q}}{\left( \left( \hat{Q}+\frac{\hat{y}_{1,3}}{2} \right)^{2}+1 \right)\left( \left( \hat{Q}-\frac{\hat{y}_{1,3}}{2} \right)^{2}+1 \right)}-\rho=0$ (A148)

For which

$\hat{y}_{1,3}=-2\sqrt{\hat{Q}^{2}-1+2\sqrt{\hat{Q}\left( \frac{\varphi}{2\rho}-\hat{Q} \right)}}$ (A149)

has already been shown to be a solution (Eqs. A47-A62), so this is also a solution for $\hat{y}_{1,3}$.

$\varphi\frac{2\hat{\delta}_{1}-\hat{y}_{1,3}}{\left( {\hat{\delta}_{1}}^{2}+1 \right)\left( \left( \hat{\delta}_{1}-\hat{y}_{1,3} \right)^{2}+1 \right)}-\rho=0$ (A150)

Can be expressed as:

${\hat{y}_{1,3}}^{2}+\hat{y}_{1,3}\left( \frac{\varphi}{\rho\left( {\hat{\delta}_{1}}^{2}+1 \right)}-2\hat{\delta}_{1} \right)+\left( {\hat{\delta}_{1}}^{2}+1 \right)-\frac{2\hat{\delta}_{1}\varphi}{\rho\left( {\hat{\delta}_{1}}^{2}+1 \right)}=0$ (A151)

Which is a quadratic in $y_{1,3}$. So there remains one additional root, which we will obtain by factoring out the preceding solution. First, we selectively substitute for:

$\hat{\delta}_{1}=\hat{Q}+\frac{\hat{y}_{1,2}}{2}$ (A152)

Which leaves us

${\hat{y}_{1,3}}^{2}+\hat{y}_{1,3}\left( \frac{\varphi}{\rho\left( {\hat{\delta}_{1}}^{2}+1 \right)}-2\hat{Q}-\hat{y}_{1,2} \right)+\left( {\hat{\delta}_{1}}^{2}+1 \right)-\frac{2\hat{\delta}_{1}\varphi}{\rho\left( {\hat{\delta}_{1}}^{2}+1 \right)}=0$ (A153)

Let A be such that:

$\left( \hat{y}_{1,3}-\hat{y}_{1,2} \right)\left( \hat{y}_{1,3}-A \right)={\hat{y}_{1,3}}^{2}+\hat{y}_{1,3}\left( \frac{\varphi}{\rho\left( {\hat{\delta}_{1}}^{2}+1 \right)}-2\hat{Q}-\hat{y}_{1,2} \right)+\left( {\hat{\delta}_{1}}^{2}+1 \right)-\frac{2\hat{\delta}_{1}\varphi}{\rho\left( {\hat{\delta}_{1}}^{2}+1 \right)}$ (A154)

$-A-\hat{y}_{1,2}=\frac{\varphi}{\rho\left( {\hat{\delta}_{1}}^{2}+1 \right)}-2\hat{Q}-\hat{y}_{1,2}$ (A155)

$A=2\hat{Q}-\frac{\varphi}{\rho\left( {\hat{\delta}_{1}}^{2}+1 \right)}$ (A156)

Therefore

$\hat{y}_{1,3}=2\hat{Q}-\frac{\varphi}{\rho\left( {\hat{\delta}_{1}}^{2}+1 \right)}$ (A157)

Is the third solution to Eq. A141.

*Assessing stability of longitudinally-coupled patches*

To assess the stability of equilibrium depth differences, we take the derivative of Eq. A141, which is:

$\frac{dD_{3}}{dy_{1,3}}=\varphi\frac{2\left( \hat{\delta}_{1}-\hat{y}_{1,3} \right)}{\left( \left( \hat{\delta}_{1}-\hat{y}_{1,3} \right)^{2}+1 \right)^{2}}-\rho$ (A158)

Equilibria are stable when:

$\frac{dD_{3}}{d\hat{y}_{1,3}}<0$ (A159)

When $y_{1,3}=0,$

${\frac{dD_{3}}{d\hat{y}_{1,3}}}_{y_{1,3}=0}=\varphi\frac{2\hat{\delta}_{1}}{\left( {\hat{\delta}_{1}}^{2}+1 \right)^{2}}-\rho$ (A160)

Given

$A=2\hat{Q}-\frac{\varphi}{\rho\left( {\hat{\delta}_{1}}^{2}+1 \right)}$ (A161)

It follows that

$\frac{2\varphi\hat{\delta}_{1}}{\rho}-\left( {\hat{\delta}_{1}}^{2}+1 \right)^{2}=\frac{\varphi}{\rho}\hat{y}_{1,2}-2\hat{Q}\hat{y}_{1,2}\left( {\hat{\delta}_{1}}^{2}+1 \right)=-A\hat{y}_{1,2}\left( {\hat{\delta}_{1}}^{2}+1 \right)$ (A162)

From which we have that

${\frac{dD_{3}}{dy_{1,3}}}_{y_{1,3}=0}=\varphi\frac{2\hat{\delta}_{1}}{\left( {\hat{\delta}_{1}}^{2}+1 \right)^{2}}-\rho=\hat{y}_{1,2}\frac{-\rho A}{\left( {\hat{\delta}_{1}}^{2}+1 \right)^{2}}$ (A163)

And is therefore negative when $A>0$. The equilibrium at $\hat{y}_{1,3}=0$ is stable whenever $\hat{\delta}_{1}<\hat{\delta}_{3}$, and therefore whenever it exists.

To assess stability of other equilibria, we first expand Eq. A141:

$-\frac{2\varphi y_{1,3}}{\rho}+\frac{2\varphi\hat{\delta}_{1}}{\rho}-\left( {\hat{\delta}_{1}}^{2}+1 \right)^{2}+2\left( {\hat{\delta}_{1}}^{2}+1 \right)\left( 2\hat{\delta}_{1}y_{1,3}-{y_{1,3}}^{2} \right)-\left( 2\hat{\delta}_{1}y_{1,3}-{y_{1,3}}^{2} \right)^{2}<0$

(A164)

And substitute using Eq. A156

$-Ay\left( {\hat{\delta}_{1}}^{2}+1 \right)-\frac{2\varphi y_{1,3}}{\rho}+2\left( {\hat{\delta}_{1}}^{2}+1 \right)\left( 2\hat{\delta}_{1}y_{1,3}-{y_{1,3}}^{2} \right)-\left( 2\hat{\delta}_{1}y_{1,3}-{y_{1,3}}^{2} \right)^{2}<0$ (A165)

When $\hat{y}_{1,3}=\hat{y}_{1,2}$,

${\frac{d\dot{D}_{3}}{dy_{1,3}}}_{y_{1,3}=\hat{y}_{1,2}}=-A\hat{y}_{1,2}\left( {\hat{\delta}_{1}}^{2}+1 \right)-\frac{2\varphi\hat{y}_{1,2}}{\rho}+2\left( {\hat{\delta}_{1}}^{2}+1 \right)\left( 2\hat{Q}\hat{y}_{1,2} \right)-\left( 2\hat{Q}\hat{y}_{1,2} \right)^{2}$ (A166)

Substituting based on Eq. A156, we have that:

${\frac{d\dot{D}_{3}}{dy_{1,3}}}_{y_{1,3}=\hat{y}_{1,2}}=A\hat{y}_{1,2}\left( {\hat{\delta}_{1}}^{2}+1 \right)-\left( 2\hat{Q}\hat{y}_{1,2} \right)^{2}<0$ (A167)

Which is true when:

$A\left( {\hat{\delta}_{1}}^{2}+1 \right)>4\hat{Q}^{2}\hat{y}_{1,2}$ (A168)

To evaluate this identity, we begin with

$A=2\hat{Q}-\frac{\varphi}{\rho\left( {\hat{\delta}_{1}}^{2}+1 \right)}$ (A169)

At equilibrium for $\hat{y}_{1,2}$

$\varphi\left( \frac{2\hat{Q}}{\left( {\hat{\delta}_{2}}^{2}+1 \right)\left( {\hat{\delta}_{1}}^{2}+1 \right)} \right)-\rho=0$ (A170)

Which can also be expressed as

$\frac{\varphi}{\rho}=\frac{\left( {\hat{\delta}_{2}}^{2}+1 \right)\left( {\hat{\delta}_{1}}^{2}+1 \right)}{2\hat{Q}}$ (A171)

Eq. A156 is therefore equivalent to:

$A=2\hat{Q}-\frac{\left( {\hat{\delta}_{2}}^{2}+1 \right)}{2\hat{Q}}$ (A172)

Since

${\hat{\delta}_{2}}^{2}+1=-\hat{Q}\hat{y}_{1,2}+2\hat{Q}^{2}+2\sqrt{\hat{Q}\left( \frac{\varphi}{2\rho}-\hat{Q} \right)}$ (A173)

Eq. A156 is also equivalent to:

$A=2\hat{Q}-\frac{-\hat{Q}\hat{y}_{1,2}+2\hat{Q}^{2}+2\sqrt{\hat{Q}\left( \frac{\varphi}{2\rho}-\hat{Q} \right)}}{2\hat{Q}}$ (A174)

and

$A=\hat{Q}+\frac{\hat{y}_{1,2}}{2}-\frac{\sqrt{\hat{Q}\left( \frac{\varphi}{2\rho}-\hat{Q} \right)}}{\hat{Q}}$ (A175)

If we assume

$A>\hat{y}_{1,2}$ (A176)

Then it follows that

$\hat{Q}-\frac{\sqrt{\hat{Q}\left( \frac{\varphi}{2\rho}-\hat{Q} \right)}}{\hat{Q}}<\frac{\hat{y}_{1,2}}{2}$ (A177)

$2\hat{Q}^{2}<\hat{Q}\hat{y}_{1,2}+2\sqrt{\hat{Q}\left( \frac{\varphi}{2\rho}-\hat{Q} \right)}$ (A178)

$4\hat{Q}^{2}<\hat{Q}\hat{y}_{1,2}+2\hat{Q}^{2}+2\sqrt{\hat{Q}\left( \frac{\varphi}{2\rho}-\hat{Q} \right)}$ (A179)

and therefore that

${\hat{\delta}_{1}}^{2}+1>4Q^{2}$ (A180)

Since $\hat{y}_{1,2}<0,$

$\hat{y}_{1,2}\left( A\left( {\hat{\delta}_{1}}^{2}+1 \right)-4Q^{2}\hat{y}_{1,2} \right)<0$ (A181)

Will be true whenever $\hat{y}_{1,2}<A$. The equilibrium where $\hat{y}_{1,3}=\hat{y}_{1,2}$ will therefore be stable when

$\hat{y}_{1,3}<A$, in which case it is the deepest of the three equilibria.

To assess stability of the third equilibrium ($\hat{y}_{1,3}=A)$, we again begin by expanding Eq. A141 to:

$-\frac{2\varphi y_{1,3}}{\rho}+\frac{2\varphi\hat{\delta}_{1}}{\rho}-\left( {\hat{\delta}_{1}}^{2}+1 \right)^{2}+2\left( {\hat{\delta}_{1}}^{2}+1 \right)\left( 2\hat{\delta}_{1}y_{1,3}-{y_{1,3}}^{2} \right)-\left( 2\hat{\delta}_{1}y_{1,3}-{y_{1,3}}^{2} \right)^{2}<0$

(A182)

And substitute in based on Eq. A156.

$-A\hat{y}_{1,2}\left( {\hat{\delta}_{1}}^{2}+1 \right)+\frac{2\varphi y_{1,3}}{\rho}+2\left( {\hat{\delta}_{1}}^{2}+1 \right)\left( 2\hat{\delta}_{1}y_{1,3}-{y_{1,3}}^{2} \right)-\left( 2\hat{\delta}_{1}y_{1,3}-{y_{1,3}}^{2} \right)^{2}<0$

(A183)

We then substitute $A$ for $\hat{y}_{1,3}$to obtain:

$-A\hat{y}_{1,2}\left( {\hat{\delta}_{1}}^{2}+1 \right)-\frac{2\varphi A}{\rho}+2A\left( {\hat{\delta}_{1}}^{2}+1 \right)\left( 2\hat{\delta}_{1}-A \right)-A^{2}\left( 2\hat{\delta}_{1}-A \right)^{2}<0$ (A184)

Which can be simplified to:

$A\left( \hat{y}_{1,2}\left( {\hat{\delta}_{1}}^{2}+1 \right)-A\left( 2\hat{\delta}_{1}-A \right)^{2} \right)<0$ (A185)

Since

$A=Q+\frac{\hat{y}_{1,2}}{2}-\frac{\sqrt{\hat{Q}\left( \frac{\varphi}{2\rho}-\hat{Q} \right)}}{Q}$ (A186)

And

$2\hat{\delta}_{1}-y_{1,3}=Q+\frac{\hat{y}_{1,2}}{2}+\frac{\sqrt{\hat{Q}\left( \frac{\varphi}{2\rho}-\hat{Q} \right)}}{Q}$ (A187)

We also have that

$\left( 2\hat{\delta}_{1}-A \right)^{2}={\hat{\delta}_{1}}^{2}+2\hat{\delta}_{1}\frac{\sqrt{\hat{Q}\left( \frac{\varphi}{2\rho}-\hat{Q} \right)}}{Q}+\frac{\hat{Q}\left( \frac{\varphi}{2\rho}-\hat{Q} \right)}{Q^{2}}$ (A188)

If $A>0$, then since $\hat{y}_{1,2}<0$, it follows that

$\hat{y}_{1,2}\left( {\hat{\delta}_{1}}^{2}+1 \right)-A\left( 2\hat{\delta}_{1}-A \right)^{2}<0$ (A189)

And

$A\left( \hat{y}_{1,2}\left( {\hat{\delta}_{1}}^{2}+1 \right)-A\left( 2\hat{\delta}_{1}-A \right)^{2} \right)<0$ (A190)

The equilibrium therefore would be stable; however, since $\hat{y}_{1,2}<0$ and $A>0$, then $\hat{\delta}_{1}<\hat{\delta}_{3}$. Therefore the assumption that $\hat{\delta}_{1}$ controls water levels would not hold, and this solution is invalid.

However, for $A<0$

$A\left( \hat{y}_{1,2}\left( {\hat{\delta}_{1}}^{2}+1 \right)-A\left( 2\hat{\delta}_{1}-A \right)^{2} \right)<0$ (A191)

Is true if

$\hat{y}_{1,2}\left( {\hat{\delta}_{1}}^{2}+1 \right)-A\left( 2\hat{\delta}_{1}-A \right)^{2}>0$ (A192)

Which can also be expressed as

$\hat{y}_{1,2}>\frac{A\left( 2\hat{\delta}_{1}-A \right)^{2}}{\left( {\hat{\delta}_{1}}^{2}+1 \right)}$ (A193)

Given $\hat{y}_{1,2}>A$, it follows that

$2\hat{Q}+\hat{y}_{1,2}-A>2\hat{Q}$ (A194)

And therefore

$\left( 2\hat{\delta}_{1}-A \right)^{2}>4\hat{Q}^{2}$ (A195)

Given $A<\hat{y}_{1,2}$, it also follows that

$Q-\frac{\sqrt{\hat{Q}\left( \frac{\varphi}{2\rho}-\hat{Q} \right)}}{Q}<\frac{\hat{y}_{1,2}}{2}$ (A196)

From which we can obtain

$2\hat{Q}^{2}>Q\hat{y}_{1,2}+2\sqrt{\hat{Q}\left( \frac{\varphi}{2\rho}-\hat{Q} \right)}$ (A197)

And:

$4\hat{Q}^{2}>Q\hat{y}_{1,2}+2\hat{Q}^{2}+2\sqrt{\hat{Q}\left( \frac{\varphi}{2\rho}-\hat{Q} \right)}$ (A198)

Since

${\hat{\delta}_{1}}^{2}+1=Q\hat{y}_{1,2}+2\hat{Q}^{2}+2\sqrt{\hat{Q}\left( \frac{\varphi}{2\rho}-\hat{Q} \right)}$ (A199)

Combining Eqs. A195, A198, and A199, we have that

$\left( 2\hat{\delta}_{1}-A \right)^{2}>{\hat{\delta}_{1}}^{2}+1>0$ (A200)

Since

$0<-\hat{y}_{1,2}<-A$ (A201)

it follows that

${-A\left( 2\hat{\delta}_{1}-A \right)}^{2}>-\hat{y}_{1,2}\left( {\hat{\delta}_{1}}^{2}+1 \right)>0$ (A202)

and

$\hat{y}_{1,2}\left( {\hat{\delta}_{1}}^{2}+1 \right){-A\left( 2\hat{\delta}_{1}-A \right)}^{2}>0$ (A203)

Therefore the equilibrium at $\hat{y}_{1,3}=A$ is stable whenever $\hat{y}_{1,2}>A$.

*Longitudinal coupling downstream of slough*

We now consider a fourth patch, with depth *D_4_* that is located downstream of patch 2.

$\frac{dD_{4}}{dt}=\hat{y}_{2,4}\left( \varphi\frac{2\hat{\delta}_{2}-y_{2,4}}{\left( {\hat{\delta}_{2}}^{2}+1 \right)\left( \left( \hat{\delta}_{2}-\hat{y}_{2,4} \right)^{2}+1 \right)}-\rho\right)$ (A203)

Which is entirely equivalent to Eq. A141. Therefore equilibria exist at:

$\hat{y}_{2,4}=0$ (A204)

$\therefore\hat{\delta}_{4}=\hat{\delta}_{2}$ (A205)

And where

$\varphi\frac{2\hat{\delta}_{2}-y_{2,4}}{\left( {\hat{\delta}_{2}}^{2}+1 \right)\left( \left( \hat{\delta}_{2}-\hat{y}_{2,4} \right)^{2}+1 \right)}-\rho=0$ (A206)

The latter solutions require that $\hat{\delta}_{4}<\hat{\delta}_{2}$, in which the assumption that $\hat{\delta}_{1}$ and $\hat{\delta}_{2}$ control water levels would not hold, and this solution is invalid. The trivial solution is therefore the only stable solution (i.e., downstream of a slough only another slough is stable).
